# Epigenetic analysis of regulatory T cells using multiplex bisulfite sequencing

**DOI:** 10.1002/eji.201545646

**Published:** 2015-10-01

**Authors:** Daniel B. Rainbow, Xin Yang, Oliver Burren, Marcin L. Pekalski, Deborah J. Smyth, Marcus D. R. Klarqvist, Christopher J. Penkett, Kim Brugger, Howard Martin, John A. Todd, Chris Wallace, Linda S. Wicker

**Affiliations:** ^1^ JDRF/Wellcome Trust Diabetes and Inflammation LaboratoryDepartment of Medical GeneticsNational Institute for Health Research (NIHR) Cambridge Biomedical Research CentreCambridge Institute for Medical ResearchUniversity of CambridgeCambridge Biomedical Research CampusCambridgeUK; ^2^ Department of HaematologyUniversity of CambridgeLong Road CambridgeUK; ^3^ East Anglian Medical Genetics ServiceMolecular Genetics LaboratoriesLevel 6 Addenbrooke's Treatment CentreAddenbrooke's HospitalCambridgeUK; ^4^ MRC Biostatistics UnitCambridge Institute of Public HealthCambridgeUK

**Keywords:** Bisulfite sequencing, FOXP3, Methylation, Next‐generation sequencing, Regulatory T cell

A subset of regulatory CD4^+^ T cells (Tregs) is characterized by the stable constitutive expression of the transcription factor FOXP3. Demethylation at a conserved region within intron 1 of *FOXP3*, the Treg‐specific demethylated region (TSDR), is exclusive to this subset of Tregs: other immune cells that do not express FOXP3 or express the transcription factor transiently, such as activated effector T cells or TGF‐β‐treated CD4^+^ T cells, are methylated at the TSDR 1, 2, 3, 4. Hence, the TSDR provides a specific target to enumerate Tregs within biological samples; developing methods that enable robust Treg‐cell quantitation with small numbers of cells from clinical samples remains an important goal. Current methods to measure *FOXP3* demethylation include qPCR 5, 6 and epigenetic sequencing methylation analysis 7 of Sanger sequencing traces 8, neither of which capture the methylation information at each CpG site on a single piece of DNA, but rather produce an average methylation over the whole region or an average at each site. Although several studies have used a PCR cloning and sequencing method to assess the methylation of each CpG site within the TSDR, this is a laborious method and normally less than 50 clones per sample are analyzed 3, 9, 10.

We therefore developed a next‐generation sequencing (NGS) method to assess at single‐base resolution the methylation status of the *FOXP3* TSDR. The method, which we originally developed to genotype rs1800521 single nucleotide polymorphism in the gene *AIRE* (Methods detailed as Supporting Information), reports at single base resolution the methylation of each DNA amplicon, provides hundreds to thousands of reads per replicate and can be multiplexed to analyze several DNA regions simultaneously.

Our method uses two‐stage PCR: the first is a gene‐specific PCR with primers having an adaptor sequence that is targeted by a second round PCR, which adds a unique index sequence enabling up to 960 different samples to be processed per sequencing run. We targeted nine CpG sites within the TSDR (Supporting Information Fig. 1), which have also been used in qPCR assays 6. Our primers flank the TSDR alleviating the need for methylation‐specific primers as well as the demethylated and methylated standards required in qPCR assays. We also developed a bioinformatics processing pipeline that reads the raw output from sequencing and reports the methylation status at each CpG (https://github.com/XinYang6699/Methpup). To validate our method, we sequenced the TSDR in three CD4^+^ T‐cell subsets, Tregs and naïve and memory effector cells, which were defined by surface molecules CD4, CD25, CD127 11 and purified by flow sorting from two male and two female donors (Fig. 1, Supporting Information Fig. 2, Supporting Information Table 1). We observed a linear relationship between the proportion of Tregs and the proportion of *FOXP3* demethylation in defined mixtures of Tregs and naïve CD4^+^ T cells (r^2^ = 0.99, Supporting Information Fig. 3). Cell inputs below 2000 cells/PCR replicate showed increasing assay variation so unless cells are extremely limiting, we typically utilize 2000–4000 cells per replicate and six replicates per cell sample (Supporting Information Fig. 4).

**Figure 1:**
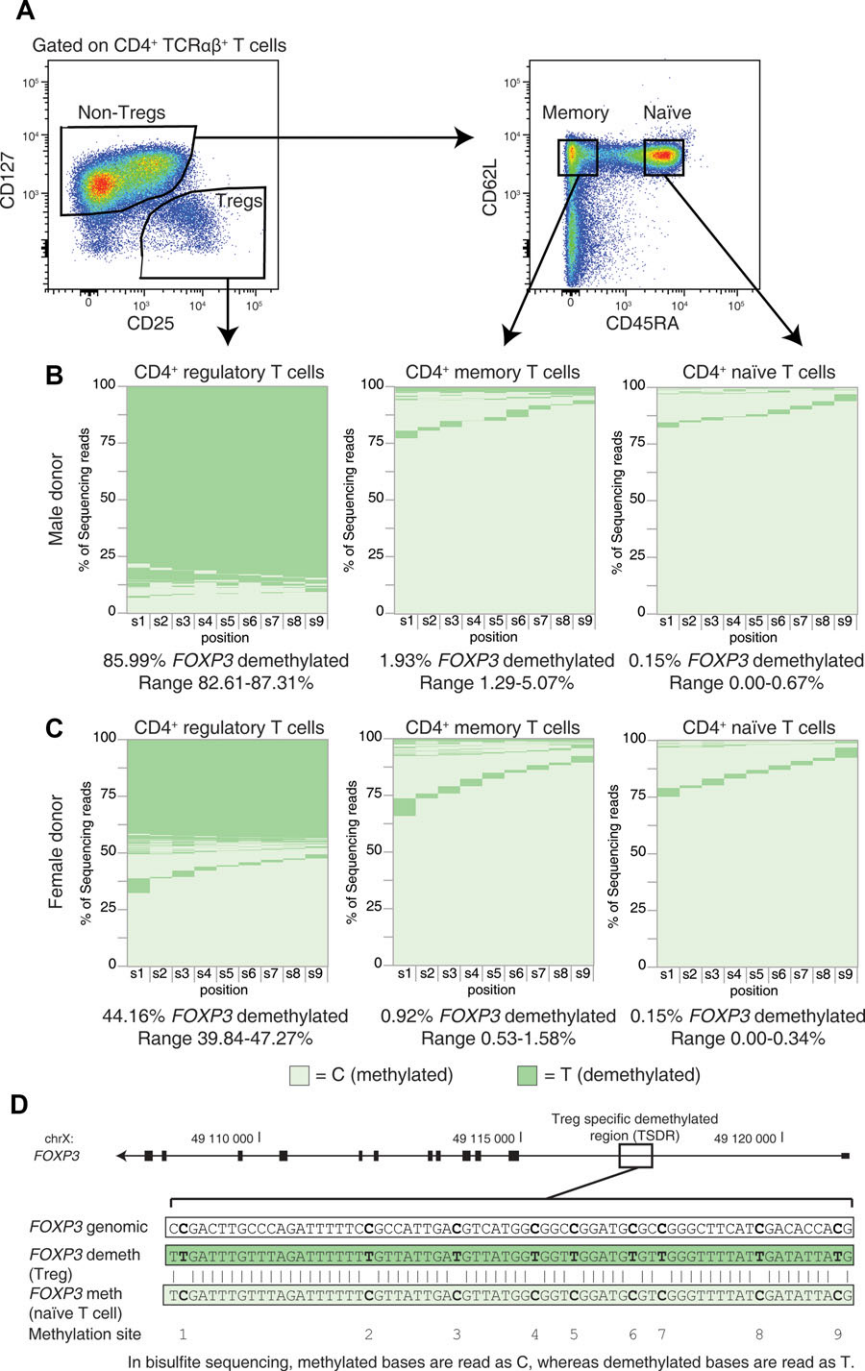
*FOXP3* TSDR sequencing of purified human CD4^+^ T‐cell subsets. (A) Naïve (CD4^+^, CD127^+^, CD45RA^+^, CD62L^+^) and memory (CD4^+^, CD127^+^, CD45RA^−^, CD62L^+^) effector cells and Tregs (CD4^+^, CD25^+^, CD127^−^) were sorted from a male (B) and a female (C) donor by flow cytometry and analyzed for *FOXP3* TSDR methylation (two representative donors out of a total of four, for more information see Supporting Information Fig. 2 and Supporting Information Table 1). See Supporting Information Fig. 7 for gating strategy defining CD4^+^ TCRαβ^+^ T cells. The x axis shows the nine methylation sites analyzed and each row indicates the methylation status of one copy of the sequenced TSDR. Light green represents a C (methylated) and dark green represents a T (demethylated). The y axis is the percentage of sequencing reads for each methylation/demethylation pattern. Six replicates of 3000 cells per replicate (5 ng DNA) were analyzed from a single donor and the median (with range) reads with eight or nine sites demethylated at *FOXP3* for the replicates is shown below each graph. (D) A schematic of the *FOXP3* gene and the TSDR sequence are shown.

As expected based on previous publications where the TSDR was shown using multiple technologies to be demethylated in a majority of Tregs 1, 3, 8, 86% and 62% of the TSDR sequences were demethylated in Treg samples from male donors and 44% and 40% from female donors (Fig. 1, Supporting Information Fig. 2, Supporting Information Table 1). Owing to X inactivation, demethylation of the *FOXP3* TSDR occurs on only one copy of the X chromosome in females thereby explaining the ∼50% lower demethylation level as compared to males. Although in Tregs the majority of highly demethylated sequencing reads are demethylated at all nine CpG sites considered, a proportion (<10%) of reads have only eight CpG sites demethylated. This incomplete demethylation could be due to biological effects or to incomplete bisulfite conversion. We favor the former possibility since bisulfite conversion efficiency as determined by cytosines that cannot be methylated was greater than 99% (Supporting Information Fig. 5A). Underscoring this biological heterogeneity, in naïve and memory effector cells demethylation of a single CpG among the nine sites was observed at each position interrogated and, when combined, comprised ∼20% of the reads. Thus the use of NGS to quantify demethylation has the advantage of revealing heterogeneity inherent in the biological process of gene regulation. As expected, and in contrast to Tregs, less than 2% of the TSDR reads from naïve and memory effector cells had a Treg‐like TSDR methylation pattern. Although this low level of Treg‐specific sequences could be caused by suboptimal cell purification, Tregs with low levels of surface CD25 and a demethylated TSDR have been reported 12 and could account for the low percentage of Treg‐specific reads observed in effector T‐cell subsets sorted by cell surface markers. The NGS‐based demethylation assay offers a great advantage in detecting small subpopulations of cells demethylated at the *FOXP3* TSDR as compared to methods that generate an average demethylation status at each residue since each NGS read simultaneously assesses the methylation status of all CpG sites from a single copy of the gene giving confidence in the interpretation of a demethylated read. Further characterization of rare subsets requires more detailed sorting in future studies including intracellular staining of FOXP3, a procedure that is compatible with NGS‐based analysis of bisulfite converted DNA 13.

Because the current version of our NGS sequencing platform utilizes up to 960 unique index sequences per sequencing run of approximately 20 million reads, each index is associated with approximately 20 000 reads. In most cases, 20 000 reads per replicate is in excess of what is required for robust measurements of percent demethylation of any particular region thereby allowing for the multiplexing of gene regions within the same sample. We used multiplexing of up to six gene regions to search for an autosomal TSDR since female samples have 50% less demethylated *FOXP3* TSDR reads than male samples. In mouse studies, specific regions in multiple genes in addition to *Foxp3* were identified that showed Treg‐specific demethylation when compared to conventional CD4^+^ T cells analyzed ex vivo: *Ctla4*, *Il2ra*, *Gitr*, *Eos*, and *Helios* 9. In comparing the human and mouse sequences in the regions reported, only one strong homology was observed, that in exon 2 of mouse *Ctla4* and human *CTLA4* (Supporting Information Fig. 6). We developed a sequencing assay for this region in the human gene, multiplexed with the *FOXP3* TSDR sequencing assay, and demonstrated that demethylation of exon 2 of human *CTLA4* is not Treg‐specific. Although >90% of Tregs analyzed ex vivo were demethylated at all seven CpG sites examined, 21–38% of memory CD4^+^ T cells were demethylated at these same sites (Fig. 2, Supporting Information Fig. 2, Supporting Information Table 2). In addition, memory effector cells displayed a heterogeneous demethylation pattern with 37–48% of reads having an intermediate methylation pattern (3T, 4T, 5T, or 6T). Thus demethylation of the human *CTLA4* exon 2 region reflects more than Treg lineage commitment.

**Figure 2:**
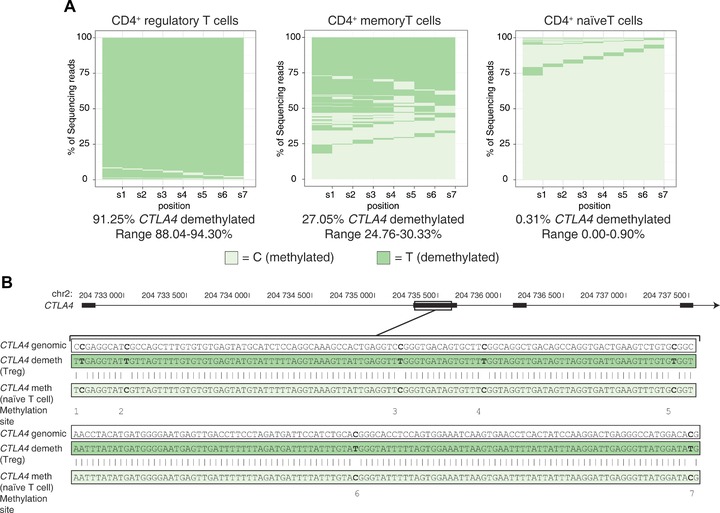
*CTLA4* demethylation in human FACS‐purified cells. Cells were purified and analyzed as detailed in Fig. 1 for *CTLA4* methylation status. The median (with range) reads with all seven sites demethylated at *CTLA4* for the replicates is shown below each graph. Data are representative of four donors (for more information see, Supporting Information Fig. 2 and Supporting Information Table 2).

To conclude, we developed a high‐throughput method for analyzing methylation at single CpG resolution that uses low DNA input and can be multiplexed. Although we have used the method to measure Treg‐specific methylation, it can be easily adapted to verify genome‐wide methylation results. Finally, given recent data showing that many disease‐associated SNPs are located in enhancer regions 14, the presence of allele‐specific methylation detected using our high‐throughput method could provide a mechanism for disease‐associated gene regulation.

## Conflict of interest

The authors declare no financial or commercial conflict of interest.

**Abbreviations**
NGS: Next‐generation sequencing
TSDR: Treg‐specific demethylated region
Treg: Regulatory T cell
qPCR: Quantitative PCR

## Supporting information

As a service to our authors and readers, this journal provides supporting information supplied by the authors. Such materials are peer reviewed and may be re‐organized for online delivery, but are not copy‐edited or typeset. Technical support issues arising from supporting information (other than missing files) should be addressed to the authors.

Epigenetic analysis of regulatory T cells using multiplex bisulfite sequencing

**Table 1**. The data that were used to generate Supporting Information Fig. 1, showing the percentage of reads with any number of CpG sites demethylated within the *FOXP3* TSDR in CD4+ naïve and memory T cells and Tregs.
**Table 2**: The data that were used to generate Supporting Information Fig. 3, showing the percentage of reads with any number of CpG sites demethylated within *CTLA4* exon 2 region in CD4+ naïve and memory T cells and Tregs
**Table 3**. A list of the 480 index sequences used. Each index was synthesized either on the forward or reverse adaptor sequence creating a total of 960 combinations
**Table 4**. Sequencing primers used in this study for methylation analysis. Lower case letters denote the adaptor sequence to which the second round index primers bind
**Figure 1**: The *FOXP3* TSDR primers were used to target ten CpG sites within the TSDR, nine of which have also been targeted by qPCR (sites numbered 1–9, Ref. 1). Site sA is not included as part of the analysis (black ovals, see Supporting Information Methods Section 1.14).
**Figure 2**: (A–C) The proportion of sequencing reads that contain the indicated number of demethylated (T) sites within each *FOXP3* read was measured from sorted Tregs and naïve and memory CD4+ T cells from two male (blue square and circle) and two female (red square and circle) donors (Supporting Information Methods Section 1.14). Supporting Information Table 1 shows percentages and read numbers. (D–F) The proportion of reads that contain the indicated number of demethylated (T) sites within each *CTLA4* read measured from sorted Tregs and naïve and memory CD4+ T cells from two male (blue square and circle) and two female (red square and circle) donors (Supporting Information Methods Section 1.14). Supporting Information Table 3 shows percentages and read numbers. The median of between four and eight PCR replicates is displayed.
**Figure 3**. 20 000 cells were sorted from total CD4+ T cells to generate differing ratios of Tregs and naïve CD4+ T cells. Six PCR replicates were performed per sample (3300 cells per replicate) and the data shown are the median with range. The Treg sample was 82.3% demethylated at the *FOXP3* TSDR, therefore the highest point graphed on the X axis.
**Figure 4**. Tregs, memory, naïve, and total CD4+ T cells were sorted and counted by the sorter and then titrated into the multiplexed bisulfite sequencing reaction. The x axis represents the number of cells used per PCR replicate. In each case, six replicates were performed from each sorted cell population. Thus, the total number of cells collected for each input is six times the number shown on the x axis. Horizontal line represents the median. Cells were not collected for the naïve and CD4+ T‐cell 250 cell point.
**Figure 5**. (A) Bisulfite conversion efficiencies for the *FOXP3* and *CTLA4* regions sequenced. (B) Relationship between cell input and quantity of bisulfite DNA extracted using the Qiagen Epitect Fast cell lysis kit (*n* = 87).
**Figure 6**: Homology between human and mouse *CTLA4* exon 2 sequences. The region identified as being differentially methylated between conventional T cells and regulatory T cells in mouse (1) was aligned to the human sequence. The CpG sites that are conserved between human and mouse are circled in red, and those that are unique to human are circled in blue. The pairwise sequence aligner, WATER (http://www.ebi.ac.uk), was used to perform the alignment.
**Figure 7**. (A) The first steps in the gating strategy used to sort Tregs, memory, and naïve CD4 T cells shown in Fig. 1. (B) Representative single parameter histograms showing the expression of FOXP3 and CTLA‐4 in Tregs, memory, and naïve CD4 T cells defined by the surface markers CD45RA, CD62L, CD127, and CD25 as shown in Fig. 1.

## References

[1] Baron, U. DNA demethylation in the human *FOXP3* locus discriminates regulatory T cells from activated FOXP3(+) conventional T cells. Eur. J. Immunol. 2007 37: 2378–2389. 17694575 10.1002/eji.200737594

[2] Floess, S. Epigenetic control of the *foxp3* locus in regulatory T cells. PLoS Biol. 2007 5: e38. 17298177 10.1371/journal.pbio.0050038 PMC1783672

[3] Miyara, M. Functional delineation and differentiation dynamics of human CD4+ T cells expressing the FoxP3 transcription factor. Immunity 2009 30: 899–911. 19464196 10.1016/j.immuni.2009.03.019

[4] Toker, A. Active demethylation of the *Foxp3* locus leads to the generation of stable regulatory T cells within the thymus. J. Immunol. 2013 190: 3180–3188. 23420886 10.4049/jimmunol.1203473

[5] Stockis, J. Comparison of stable human Treg and Th clones by transcriptional profiling. Eur. J. Immunol. 2009 39: 869–882. 19224638 10.1002/eji.200838807

[6] Wieczorek, G. Quantitative DNA methylation analysis of *FOXP3* as a new method for counting regulatory T cells in peripheral blood and solid tissue. Cancer Res. 2009 69: 599–608. 19147574 10.1158/0008-5472.CAN-08-2361

[7] Lewin, J. Quantitative DNA methylation analysis based on four‐dye trace data from direct sequencing of PCR amplificates. Bioinformatics 2004 20: 3005–3012. 15247106 10.1093/bioinformatics/bth346

[8] Spreafico, R. A sensitive protocol for *FOXP3* epigenetic analysis in scarce human samples. Eur. J. Immunol. 2014 44: 3141–3143. 25042685 10.1002/eji.201444627

[9] Ohkura, N. T cell receptor stimulation‐induced epigenetic changes and Foxp3 expression are independent and complementary events required for Treg cell development. Immunity 2012 37: 785–799. 23123060 10.1016/j.immuni.2012.09.010

[10] Kim, H. P. CREB/ATF‐dependent T cell receptor‐induced FoxP3 gene expression: a role for DNA methylation. J. Exp. Med. 2007 204: 1543–1551. 17591856 10.1084/jem.20070109 PMC2118651

[11] Liu, W. CD127 expression inversely correlates with FoxP3 and suppressive function of human CD4+ T reg cells. J. Exp. Med. 2006 203: 1701–1711. 16818678 10.1084/jem.20060772 PMC2118339

[12] Bending, D. Hypomethylation at the regulatory T cell‐specific demethylated region in CD25^hi^ T cells is decoupled from FOXP3 expression at the inflamed site in childhood arthritis. J. Immunol. 2014 193: 2699–2708. 25092890 10.4049/jimmunol.1400599 PMC4157061

[13] McClymont, S. A. Plasticity of human regulatory T cells in healthy subjects and patients with type 1 diabetes. J. Immunol. 2011 186: 3918–3926. 21368230 10.4049/jimmunol.1003099 PMC3091943

[14] Zilbauer, M. Genome‐wide methylation analyses of primary human leukocyte subsets identifies functionally important cell‐type specific hypomethylated regions. Blood 2013 122: e52–e60. 24159175 10.1182/blood-2013-05-503201 PMC3862273

