# Supplementary material for: Epigenetic analysis of regulatory T cells using multiplex bisulfite sequencing
Source: Eur J Immunol. 2015 Oct 1;45(11):3200–3. doi: 10.1002/eji.201545646 (PMC4832369; doi:10.1002/eji.201545646)
Supplement: Supplementary file 2 — Table 1. The data that were used to generate Supporting Information Fig. 1, showing the percentage of reads with any number of CpG sites demethylated within the FOXP3 TSDR in CD4+ naïve and memory T cells and Tregs. Table 2: The data that were used to generate Supporting Information Fig. 3, showing the percentage of reads with any number of CpG sites demethylated within CTLA4 exon 2 region in CD4+ naïve and memory T cells and Tregs Table 3. A list of the 480 index sequences used. Each index was synthesized either on the forward or reverse adaptor sequence creating a total of 960 combinations Table 4. Sequencing primers used in this study for methylation analysis. Lower case letters denote the adaptor sequence to which the second round index primers bind Figure 1: The FOXP3 TSDR primers were used to target ten CpG sites within the TSDR, nine of which have also been targeted by qPCR (sites numbered 1–9, Ref. 1). Site sA is not included as part of the analysis (black ovals, see Supporting Information Methods Section 1.14). Figure 2: (A–C) The proportion of sequencing reads that contain the indicated number of demethylated (T) sites within each FOXP3 read was measured from sorted Tregs and naïve and memory CD4+ T cells from two male (blue square and circle) and two female (red square and circle) donors (Supporting Information Methods Section 1.14). Supporting Information Table 1 shows percentages and read numbers. (D–F) The proportion of reads that contain the indicated number of demethylated (T) sites within each CTLA4 read measured from sorted Tregs and naïve and memory CD4+ T cells from two male (blue square and circle) and two female (red square and circle) donors (Supporting Information Methods Section 1.14). Supporting Information Table 3 shows percentages and read numbers. The median of between four and eight PCR replicates is displayed. Figure 3. 20 000 cells were sorted from total CD4+ T cells to generate differing ratios of Tregs and naïve CD4+ T cells. Six PC [file EJI-45-3200-s002.pdf]

Supporting Information

Methods

A summary of the work flow is shown in Supporting Information Fig. 8.

1.1 Subjects

Research involving human participants was approved by the NHS Cambridgeshire Research Ethics Committee. Samples were obtained from the Cambridge BioResource (www.cambridgebioresource.org.uk), as part of the 'Genes and Mechanisms of Type 1 Diabetes' study and were of self-reported white ethnicity and informed consent was obtained from all volunteers for the collection and use of the peripheral blood samples. Data and samples are always treated anonymously and confidentially.

1.2 Cell sorting

$CD4^+$ T cells were purified using RosetteSep human $CD4^+$ T-Cell Enrichment Cocktail (Stemcell Technologies) from blood. The $CD4^+$ T cells were stained with the following panel of antibodies to surface markers and $CD4^+$ naïve and memory T cells and Tregs were sorted on an BD FACSAria III: TCRαβ (FITC, clone IP26, BioLegend), CD4 (AF700, clone RPA-T4, BioLegend), CD25 (APC, clones M-A251 and 2A3, BD Biosciences, both clones were added together to increase sensitivity), CD127 (PE-Cy7, clone eBioRDR5, eBioscience), CD45RA (BV785, clone HI100, BioLegend), CD62L (eFluor605, clone DREG56, eBioscience) and Viability Dye (eFluor780, eBioscience). For intracellular staining, FOXP3 (PE, clone 259D, BioLegend) and CTLA-4 (PE-CF594, clone BNI3, BD Biosciences) antibodies were incorporated into the above panel. Supporting Information Fig. 7A exemplifies the initial gating strategy of $CD4^+$ $TCR\alpha\beta^+$ T cells and Fig. 1 shows the gating of Tregs and naïve and memory effector $CD4^+$ T cells. Sort purity was determined in a subset of samples and ranged from 98.0-99.9%. We opted for sorting Tregs using cell surface markers rather than FOXP3 primarily due to the reduction in the yields of Tregs caused by the additional washes required for the intracellular staining protocol for the transcription factor. It was shown by Bluestone and colleagues [1] that $CD4^+$ $CD25^+$ $CD127^{-/low}$ cells are highly enriched for $FOXP3^+$ cells (86.6%, range 67.4-93.6 for 10 donors – Table 1 in their paper, Ref 1) that mediate suppression (Fig. 7, Ref 1) and other investigators [2] have confirmed their observation. We have confirmed that Tregs, naïve and

memory effector T cells defined by surface markers show differential intracellular expression of FOXP3 with the greatest expression in Treg cells (Supporting Information Fig. 7B). Similarly, a strong correlation between the levels of FOXP3 and CTLA-4 in Tregs has been demonstrated by Miyara et al. [2] and we have confirmed these findings (Supporting Information Fig. 7B).

1.3 Multiplex DNA variant sequencing

rs1800521 is a synonymous SNP (Ala>Ala) located in the *AIRE* (autoimmune regulator) gene on the long arm of chromosome 21. A previous study using a pooled sequencing dataset found association with type 1 diabetes, with the minor allele frequency (MAF) in cases being 0.32 and in controls 0.23, with a Fisher's P value of 1.8×10^{-5} [3]. We had attempted to genotype rs1800521 using a Taqman genotyping assay from Applied Biosystems and Illumina Immunochip, but these methods were unsuccessful, most likely due to the surrounding sequence being GC rich. This SNP was not in high LD with any other SNP so a tag SNP could not be used. Hence, a next generation sequencing approach was developed to genotype the SNP rs1800521 in thousands of samples. We developed an index system that allows up to 960 different samples to be analysed per MiSeq sequencing run. Primers were designed that flank the SNP rs1800521 (Forward – tgtaaaacgacggccagtCCGGCATGGACACGACTCTT, reverse – caggaaacagctatgaccGTAGGTCCTGGGCTCCTTGA; lowercase letters denote the adaptor sequence to which the second round index primers bind) and a second stage PCR added the index sequence (Supporting Information Table 3) to the adaptor sequence of the first round primer (shown in lowercase). Initially we sequenced DNA samples from 48 type 1 diabetes cases and 48 control samples to confirm the method worked, and the minor allele frequencies (MAF) were 0.375 and 0.277, respectively. We then sequenced a further 2962 cases and 2777 controls and the MAF was 0.369 and 0.368, respectively. Therefore, there was no evidence of association of SNP rs1800251 with type 1 diabetes.

1.4 DNA extraction and bisulfite conversion

Samples with fewer than 100,000 cells were processed in a one step cell lysis and bisulfite conversion using Qiagen Epitect lyse all kit. For samples with greater than 100,000 cells, the DNA was first extracted using Qiagen QIAMP DNA micro kit. A maximum of 500 ng DNA was bisulfite treated using a Qiagen Epitect Fast DNA bisulfite kit. Bisulfite-converted DNA was quantified using a nanodrop with the single strand DNA setting. Supporting Information Fig. 5B shows the amount of DNA extracted from various cell inputs. For all steps lo-bind

DNA tubes (Eppendorf) were used to reduce the loss of DNA, especially from low cell input samples from adsorption onto the tube wall.

1.5 First round PCR primer design

The DNA sequence from the region of interest was downloaded from Ensembl (http://www.ensembl.org/Homo_sapiens/Info/Index) and all the C bases were changed to T, unless they were paired to a G in which case they were flanked by square brackets, such that in the primer design program, Primer 3 (http://bioinfo.ut.ee/primer3-0.4.0/primer3/), the primers were forced to surround the methylation sites. Standard primer picking conditions we used, except optimal primer length was increased to 24 bp. Adaptor sequences were added to the gene-specific primers, shown as lowercase letters (Supporting Information Table 4). For the *FOXP3* TSDR, primers were designed to capture the same nine CpG sites that were targeted by published qPCR assays [4], however due to sequence restrictions of primer design we captured an additional CpG site which was analysed (Supporting Information Fig. 1), but this site was dropped from our analysis when calculating the percent *FOXP3* demethylation as it showed an inconsistent methylation pattern (see section 1.14 Methylation-based cell quantification for more details).

1.6 First round PCR

The first round PCR contains 10 µl of Qiagen multiplex PCR mastermix, 0.5 µl of 10 nmol forward primer, 0.5 µl of 10 nmol reverse primer, 2 µl of bisulfite DNA (4-8 ng) and made up to 20 µl with ultra-pure water. The PCR cycling conditions were 95°C for 15 minutes hot start, followed by 25 cycles of the following steps: 95°C for 30 seconds, 60°C for 90 seconds and 72°C for 60 seconds, finishing with a 72°C for 10 minutes cycle. Primers used to interrogate *FOXP3* and *CTLA4* are detailed in Supporting Information Table 4. The 5′ end of the gene-specific primers are tagged with M13F and M13R sequences (shown as lowercase in Supporting Information Table 4) to which the second round PCR primers anneal. All the regions described in this paper have been multiplexed from the same sample, using 2000 – 4000 cells per reaction (4-8 ng bisulfite DNA (Supporting Information Fig. 5B)) with six independent PCR and sequencing reactions performed. This is a much more feasible range for clinical samples, compared to 30 ng per replicate per gene reported for qPCR [4]. A similar input has been used with Sanger sequencing, but has the disadvantage of not being able to multiplex PCR [5].

1.7 Ampure XP PCR cleanup

Ampure XP beads (Beckman Coulter) were used to purify the PCR products at the end of the first round PCR. To the 20 µl PCR reaction, 36 µl Ampure XP beads were added and incubated for 5 minutes. Two washes with 70% ethanol followed and the DNA was eluted in 20 µl of ultra-pure water. Only 18 µl of sample was collected to reduce the carryover of Ampure beads.

1.8 Second round PCR

To add Illumina sequence compatible ends to the individual first round PCR amplicons, additional primers were designed to incorporate P1 and A sequences plus sample-specific index sequences in the A primer, through hybridisation to the M13 tag sequences present on the first round gene-specific primers. Index sequences were designed using a bespoke Perl script to be 9 bp in length, without polynucleotide repeats and lack any significant secondary structure or homology to the P1 or A ends. The index sequences used are listed in Supporting Information Table 5.

The second round PCR contains 7.5 µl of Qiagen multiplex PCR mastermix, 2.5 µl of ultra-pure water, 0.5 µl of each forward and reverse index primer, 4.5 µl of Ampure XP-cleaned first round PCR product. The PCR cycling conditions were 95°C for 15 minutes hotstart, followed by 7 cycles of the following steps: 95°C for 30 seconds, 56°C for 90 seconds, 72°C for 60 seconds, finishing with 72°C for 10 minutes cycle.

1.9 Pooling PCR products

All PCR products were run on a Shimadzu Multina to quantify the amount of product. Equimolar amounts of PCR product were pooled into a single tube, and Ampure XP beads were used to remove unincorporated primers and to concentrate the pooled sample, which was eluted in ultra-pure water.

1.10 Library quantification

To accurately quantify the library for sequencing we used the Kapa Bioscience library quantification kit, according to manufacturer’s instructions. In brief, the library was diluted to 10 nM, based on Qubit (Life Technologies) quantification, and this was diluted 1:1000, 1:2000, 1:4000 and 1:8000, which was used as the template for qPCR run on an Applied

Biosystems 7900HT, and compared to a standard curve at dilutions from 20 pmol to 0.0002 pmol.

1.11 Sequencing

The library was sequenced on an Illumina MiSeq using either v2 reagents (2 x 250 bp reads) or, since their release in December 2013, v3 reagents (2 x 300 bp reads). PhiX was spiked into the sequencing run at 5 % to add diversity. MiSeq output using V3 reagents is approximately 25 million paired end reads per run.

1.12 Data processing

The raw FASTQ data files from the Illumina Miseq were demultiplexed using Novobarcode (http://www.novocraft.com). The forward and reverse FASTQ files were split into a unique file for each of the index sequences used (up to 960). Trimmomatic version 0.27 [6] was used to trim the adaptor sequence (HEADCROP:18), trim the end of the read when the quality score dropped below 20 (TRAILING:20) and a sliding window trimming was performed when the average base quality of four continuous bases fell below 20 (SLIDINGWINDOW:4:20). Next, Cutadapt version 1.2.1 [7] was used to remove the Illumina adaptor sequence. Given our targeted sequences are quite short (e.g. 218 bp for *FOXP3* TSDR) compared to the read length (250 bp), there are overlaps between forward and reverse reads. Therefore, they were stitched together and where the reads overlapped, the base with the higher quality score was kept using FLASH-1.2.4 [8]. Bowtie 2 [9] was used to align each stitched sequence read to our customized gene reference sequence with the options --phred33 --np 0 --no-unal --no-head --local. Indels were adjusted properly before the CpG sites were called using an in-house python script, which deletes the insertions and adds an ambiguous letter "D" if there is a deletion. The pipeline to perform all these steps is available from https://github.com/XinYang6699/Methpup. Bisulfite conversion efficiency was assessed by counting the number of C bases present outside of CpG sites, which should have been converted to T by the bisulfite treatment, and was greater than 99% (Supporting Information Fig. 5A).

1.13 Methylation profile visualization

R was used to read and visualize the processed data as a two-dimensional barcode. Our visualization functions are available in the R package: Methplot (http://cran.r-project.org/web/packages/Methplot/index.html).

1.14 Methylation-based cell quantification

In calculating the percentage of TSDR reads that are demethylated, the most proximal CpG site (called sA in Supporting Information Fig. 1) was excluded from our analysis as it showed a demethylation pattern not consistent with the other nine CpG sites analysed, which has been reported previously [4, 5, 10] and the proportion of demethylation was greater in DNA from females (Supporting Information Fig. 1). We classified Tregs as having 8 or 9 of the CpGs sequenced demethylated, as when we counted the numbers of reads with 0, 1, 2, ……, 9 sites demethylated we see that in sorted Tregs there are some reads with 8 sites and most reads with 9 sites demethylated, but very few reads with between 3 and 7 CpG sites demethylated (Supporting Information Fig. 2A). In sorted naïve and memory $CD4^+$ effector T cells, the opposite pattern is seen with >97.7% of the reads having 0, 1 or 2 CpG sites demethylated (Supporting Information Fig. 2B, C). The same analysis was performed for *CTLA4* (Supporting Information Fig. 2D-F and Supporting Information Table 2), and we found that although Tregs were preferentially demethylated at all seven CpGs (>90% of sorted Tregs), ~30% of memory cells and ~5% of naïve cells were demethylated to this extent.

1.15 Cell input titration

To determine the lower input level for the multiplexed bisulfite sequencing assay, Tregs, memory and naïve $CD4^+$ T cells as well as total $CD4^+$ T cells were sorted. To increase the accuracy of the experiment, the BD FACSAria III sorter counted and collected between 60,000 and 750 cells per tube from each of the four cell populations and bisulfite DNA was prepared. Six PCR replicates were performed per sample so that bisulfite DNA prepared from a maximum of 10,000 and a minimum of 125 cells was used as the template for the multiplexed bisulfite sequencing reaction. The results show that between 2,000 and 6,000 cells there was very little difference in the levels of methylation, however below 2,000 cells per PCR replicate, variation amongst the replicates increased, which was especially noticeable when low levels of demethylation were present (Supporting Information Fig. 4). The 10,000 cells per PCR replicate also had some variation of replicates most likely due to overloading the PCR reaction.

1.16 Methylation-based cell quantification

To assess the accuracy of the multiplexed bisulfite sequencing assay, various proportions of Tregs and naïve $CD4^+$ T cells were sorted using the BD FACSAria III so that in total 20,000

cells were collected per point. Bisulfite DNA was made from the cells collected and six PCR replicates were performed per sample so that 3,300 cells were used per PCR reaction. The 100% Treg sample had 82.3% reads demethylated at the *FOXP3* TSDR. The data demostrate that for both the *CTLA4* exon 2 (r^2=0.998) and the *FOXP3* TSDR (r^2=0.994) sequencing assay there is linearity at all points analysed (Supporting Information Fig. 3).

References

1 **Liu, W.** *J Exp Med* 2006. **203**: 1701-1711.

2 **Miyara, M.** *Immunity* 2009. **30**: 899-911.

3 **Yang, X.** *Bioinformatics* 2012. **28**: 2898-2904.

4 **Wieczorek, G.** *Cancer Res* 2009. **69**: 599-608.

5 **Spreafico, R.** *Eur J Immunol* 2014. **44**: 3141-3143.

6 **Bolger, A. M.** *Bioinformatics* 2014. **30**: 2114-2120.

7 **Martin, M.** *EMBnet.journal* 2011. **17**: 10-12.

8 **Magoc, T.** *Bioinformatics* 2011. **27**: 2957-2963.

9 **Langmead, B.** *Nat Methods* 2012. **9**: 357-359.

10 **Baron, U.** *Eur J Immunol* 2007. **37**: 2378-2389.

Supplemental table 1: The data that were used to generate Supplemental figure 1, showing the percentage of reads with any number of CpG sites demethylated within the *FOXP3* TSDR in CD4$^+$ naïve and memory T cells and Tregs.

Sample	Sex	9T	8T	7T	6T	5T	4T	3T	2T	1T	0T	% Tregs	Read number
Tregs*	Female	41.81	2.50	0.60	0.38	0.22	0.39	1.21	4.60	16.25	32.90	88.31 (79.68-94.54)	27803
Tregs	Female	38.35	1.58	0.22	0.04	0.07	0.07	0.42	3.49	15.60	39.95	80.55 (56.49-89.24)	110083
Tregs*	Male	76.83	9.22	1.17	0.18	0.04	0.07	0.67	1.08	3.14	7.01	85.99 (82.61-87.31)	25184
Tregs	Male	60.11	1.91	0.02	0.14	0.03	0.00	0.08	3.53	7.09	29.53	61.83 (51.20-76.41)	6003
Memory T cells*	Female	0.75	0.16	0.10	0.04	0.17	0.18	0.96	5.33	23.91	67.81	1.84 (1.06-3.16)	29330
Memory T cells	Female	0.12	0.00	0.00	0.00	0.02	0.04	0.43	2.81	22.22	74.59	0.24 (0.00-0.96)	118935
Memory T cells*	Male	1.46	0.17	0.18	0.03	0.00	0.15	0.61	2.15	18.46	74.15	1.93 (1.29-5.07)	23308
Memory T cells	Male	1.50	0.00	0.00	0.00	0.00	0.00	0.00	1.75	18.92	76.38	1.50 (0.44-2.35)	3244
Naïve T cells*	Female	0.12	0.01	0.00	0.00	0.00	0.00	0.29	2.96	20.74	76.02	0.29 (0.00-0.49)	25398
Naïve T cells	Female	0.00	0.00	0.00	0.00	0.00	0.00	0.08	1.92	18.57	79.35	0.00 (0.00-0.05)	92288
Naïve T cells*	Male	0.14	0.00	0.00	0.00	0.00	0.00	0.11	1.84	18.52	79.55	0.15 (0.00-0.67)	26146
Naïve T cells	Male	1.23	0.00	0.00	0.00	0.00	0.00	0.00	1.61	20.21	76.65	1.68 (0.00-2.24)	4958
**Comments**			**A**							**B**		**C**	**D**

A = Reads with 8 CpGs demethylated represent the laddering pattern seen in CD4$^+$ Tregs (Fig 1B and C left hand panel).

B = Reads with 1 CpG site demethylated represents the laddering pattern seen in non-Tregs (naïve and memory CD4$^+$ T cells, Fig 1B and C right hand panels).

C = Percentage Treg calculated as the number of sequencing reads with 8 or 9 CpG sites demethylated divided by the total number of reads. For samples from female donors, the percent Treg value has been multiplied by two, to account for X chromosome inactivation. Between 4 and 8 PCR replicates was performed per sample and the data shown is the median (with range).

D = The read number is the sum of the reads analysed from all PCR replicates. The number of reads obtained per sample is dependent on the number of samples analysed and the number of genes multiplexed per sequencing run.

* = Indicates the samples that are depicted in Figure 1.

Supplemental table 2: The data that was used to generate Supplemental figure 3, showing the percentage of reads with any number of CpG sites demethylated within *CTLA4* exon 2 region in CD4$^+$ naïve and memory T cells and Tregs.

Sample	Sex	7T	6T	5T	4T	3T	2T	1T	0T	Read number
Tregs	Female	90.61 (89.65-91.97)	6.78	1.03	0.72	0.43	0.22	0.15	0.36	6756
Tregs	Female	93.04 (89.39-96.01)	5.98	0.14	0.06	0.01	0.00	0.04	0.00	44362
Tregs*	Male	91.25 (88.04-94.30)	7.14	0.75	0.00	0.00	0.00	0.00	0.10	8601
Tregs	Male	92.30 (80.33-98.25)	2.87	0.20	1.14	0.00	0.20	0.69	0.00	1368
Memory T cells	Female	21.43 (20.59-23.21)	11.20	10.55	8.56	9.17	9.82	13.26	14.25	8243
Memory T cells	Female	28.20 (22.60-34.33)	13.62	10.33	7.90	8.98	9.31	10.27	11.43	49423
Memory T cells*	Male	27.05 (24.76-30.33)	16.99	11.80	9.83	8.94	9.07	8.51	7.19	6217
Memory T cells	Male	37.97 (26.99-48.43)	14.56	10.18	4.72	8.18	6.64	9.49	5.66	860
Naïve T cells	Female	0.06 (0-0.19)	0.07	0.24	0.71	0.84	4.10	21.54	71.60	13796
Naïve T cells	Female	0.00 (0.00-0.00)	0.00	0.00	0.00	0.25	2.24	14.59	81.94	14353
Naïve T cells*	Male	0.31 (0-0.90)	0.23	0.65	1.10	1.17	5.17	19.86	71.26	10207
Naïve T cells	Male	4.87 (0.70-9.02)	5.14	0.51	1.88	0.00	2.10	12.67	73.98	1253
**Comments**		**A**								**B**

A = The percentage of reads which have all seven CpG sites within the *CTLA4* exon 2 region that are fully demethylated, data is the median with range.

B = The read number is the sum of the reads analysed from all PCR replicates. The number of reads obtained per sample is dependent on the number of samples analysed and the number of genes multiplexed per sequencing run.

* = Indicates the samples depicted in Figure 2.

Supplemental table 3:
A list of the 480 index sequences used. Each index was synthesised either on the forward or reverse adaptor sequence creating a total of 960 combinations.

**Index sequence**	**Index sequence**	**Index sequence**	**Index sequence**
ACACGAGGA	GACAGTGAG	AATTAGCGT	CCCAGGTAG
ACAGCTTGA	GACTGAGTC	AATTGCAGT	CAAGAGAGT
ACATATGCG	GAGAGCTTC	AAATACGCT	CATTGGAAT
ACGACTAAT	GAGCGCTAT	AATGGATAG	CACATACGA
ACGTCGCAG	GAGTGAGAT	AACAAAGGC	CTAAGTCTC
ACTAGTCAG	GATATAGTC	AACTGGTGC	CTTCAATCA
ACTCCGTAT	GATCTAGAT	ATAATTCAG	CTGATCGTT
ACTGTGATG	GATGCTGCG	ATTGACCGA	CTCACGCTA
AGACTTGCA	GCACTAGAC	ATCAGACGC	TAAACTACA
AGAGATATG	GCAGTCGAT	AGAACTGCC	TAATACTTC
AGATAGGTA	GCATATATA	AGTACGCAC	TTGAGGTGC
AGCAGTCAC	GCGAGAGAC	AGGCGCGTC	TGGATAATC
AGCTGCATA	GCGTCGAAT	AGCGAGTTC	TCTACAATT
AGTACGATT	GCTAGATGC	ACACTCCAT	TCGCACCTA
AGTCGTAGC	GCTCTTGTG	ACTTCGGAA	TCCCAACAT
AGTGACGTC	GCTGTGCAG	ACGTGCTGG	TAAGATCAC
ATACGTATA	GTACGTAGC	ACCTACCTG	TTCACCAGT
ATAGTGAAG	GTAGTACGC	AATTCCTCA	TGCTACTTA
ATATCGCTC	GTATACGCA	AAACTAAGG	TCAGCAGGT
ATCACATGG	GTCATGCAA	AATCGCTCT	TCTTCGAGT
ATCTAGTGG	GTCTATCTA	AAGCCTATG	TCGTAGTTC
ATGAGATGA	GTGAGTGTC	AACGGCCTG	TCCTCCAGA
ATGCAGCAC	GTGCTGATA	ATAAGCTGT	GAAAGTATG
ATGTGCTCT	GTGTAGTCA	ATTCATGTT	GAATCTTAA
CACAGACCG	TACACTCAC	ATGCAGAGG	GATTATGTA
CACTAGATG	TACTCATGC	ATCGCATTC	GAGGCCAGG
CAGACAGAG	TAGATCAGT	AGCACTAGC	GACGAACCT
CAGCAAGTC	TAGCGTAAT	AGGAGAGTT	GTACGGTAC
CAGTCTACT	TAGTCACGA	AGGTGTGGA	GTTCGACTT
CATACGATG	TATATCTAC	ACAATCCTC	GTGACTAGC
CATCTATGC	TATCAGAGT	ACTCCCTTC	GTCACCAAA
CATGTCTTA	TATGACATA	ACGGCACTG	GTCTCATGT
CGACATAGC	TCACATTCA	ACCCATATC	GGAGATAAC
CGAGCGCGT	TCAGTGCTT	CAAATCTGG	GGTCATTGA
CGATCTCTG	TCATATCTG	CATAGTCGA	GGGAGAGGA
CGCAGTGAT	TCGAACTAG	CAGCGGCTA	GGGTACACT
CGCTATCAT	TCGTATGAG	CACCTCCTT	GGCGCGTCG
CGTATCATC	TCTAGCAAG	CTAGTAGTG	GCACCAGCA
CGTCTGTAT	TCTCAGATC	CTTGTAATT	GCTCACATT
CGTGTAGAC	TCTGAAGAT	CTGGATACA	GCGAATCGT
CTACTCAGT	TGACGTACA	CTCCAGTAT	GCGTACCGA
CTAGCATGA	TGAGTCAAT	CGACACTGG	GAAGATCTT
CTATGCTAC	TGATGCATG	CGTGATCAA	GATCATCCT
CTCAGTAGA	TGCATTCGT	CGGGAGCCT	GAGCATTCA
CTCTGCACA	TGCTTAGTC	CGCGTCACT	GACCAGATC
CTGATGAAT	TGTATGATA	CCAGCAACG	GTAAGATCT
CTGCTGTGC	TGTCGCGAG	CCTCTCAGA	GTTAACCTC
CTGTAGAGC	TGTGTAGTG	CCGCATATT	GTTTCACTA
ATGACTTCC	CCATGCCCG	CGGCAACCA	CTTAGCTCG
ATTCACTAT	CCCAGAGGC	CGGCGACAC	CTTCAGAAG
CAAACTTCC	CCCTCAAAT	CGGTTCAGG	CTTGCTCCT
CAAGAATTC	CCGATCTGC	CGTACTGAA	GAACGAACG
CAATCAGCA	CCGGATCAG	CGTCATTAC	GACACTGGT
CACCAGCAG	CCTCAGCGT	CTACGGCCT	GAGATGCCT
CACTCTCTC	CCTTAGCTG	CTCAGCATT	GATAGCCTA
CAGAGTTCA	CGAACGGTT	CTCCACATG	GCAATACCA
CAGTGGTGT	CGAATCTCT	CTCTCAACG	GCAGAGCAA
CATTCAGTC	CGATAGTGC	CTGAGTTAG	GGCTCAATA
CCAACTTGT	CGCCAATAA	CTGCGGGAC	GGGCGGTTC
CCACAATCT	CGCCAGGAC	CTGCTTAAC	GGGGATGTA

Index sequence	Index sequence	Index sequence	Index sequence
AATTAGTTG	CCCGACCCA	AATTATCAG	CAGAGTATT
AATTGGGTT	CGTAACGCG	AATTGTTGA	CACATCAAG
AAATCTGTA	CGGAGCCTA	AATATACGC	CTAATCACC
AAGACCTGC	CGCACGGCG	AAGAGACGT	CCGACAGCA
AACATGTGT	CCAATACAG	AACGATGCC	CCGTATCGC
ATAACCAAC	CCTACACTG	ATAACGGGG	CCCTGCGTG
ATTACAAGC	CCTTATGGT	ATTACACTT	CTCTCTGAG
ATTGCGTGA	CCGTAGGAT	ATGAACTAC	CCCTGACTA
ATCATCTCA	TAAAGCAGA	ATCCGCGAG	CCTATACGT
AGAATGTTC	TTATATACC	AGATTGTGT	TAACCTTGC
AGTCAGGCT	TTGATACGG	AGTCCTCAT	TTATATTAT
AGGGGCAGT	TCAAGAATC	AGGGTACCT	TGAGCTCTT
AGCGTCTAA	TCTCAGTCG	AGCTATAGT	TCAAGTAGT
ACAGACCTA	TCGCACGCT	ACAGAGTCC	TCTGCTGGC
ACGAGGTCT	TCCCATACG	ACGCACCAG	TCGGAACAC
ACCAAGCCG	TAAGCACCA	ACCAGGAGA	TCCGAATTC
ACCTAGACA	TTCATAGTT	AAAGGACCG	TAATACACA
AATTCTCGC	TGCTGAAGC	AATGATTAT	TTCGACCTG
AAAGCATCT	TCAGTGGAC	AAGTCACAT	TGGAGACCT
AATCGTGAT	TCTTGCGAT	AACTCAGTG	TCATCATTG
AAGTAGGAC	TCGTGATCT	ATAATGTAT	GAACATAGA
AACGGTCGT	TCCTCGTAG	ATTCTACAT	GATACACCA
ATAAGGACA	GAAATCACT	ATGGCGGCC	GAGAACTAA
ATTCGAGGC	GAATGTACT	ATCTAAGAC	GACACGAGA
ATGGAATAG	GATTTCAAA	AGTAATCGT	GACTGCCAG
ATCGTTAGG	GAGTTAACT	AGGCAGAAT	GTATCGTGA
AGCAGGATG	GACGACTAC	AGCCTTCTG	GTTGATATA
AGGAGTACG	GTAGACCGT	ACACGTCGG	GTGCACGGC
AGCCTAAGC	GTTGACAAT	ACTTAACAA	GTCCAATTC
ACAATTTCT	GTGCAAGCT	ACGTCTATC	GGAACGACA
ACTGACTCT	GTCACCGTC	ACCGTGTGG	GGATACGTC
ACGGCATGT	GTCTCGCAC	CAACGAGAG	GGTGACCCA
ACCGTACCC	GGAGATTGG	CATGCCTAT	GGGCCTTCT
CAACAACAT	GGTCTAGTA	CAGGTTCCC	GGCAGGCTC
CATCTGGCC	GGGAGGCAT	CACGGTGTG	GGCTACTGG
CAGGCAATC	GGGTACGAA	CTTAGACCT	GCAGAACTC
CACGCTGCT	GGCGCTCAA	CTTTATACG	GCTGCAATA
CTATCGACT	GCACGACAT	CTGTGATTC	GCGCAGCCG
CTTTACGTA	GCTCCAGAG	CTCGCTAAT	GAAGCTGGA
CTGGTGGCA	GCGACACGC	CGAGGTGTT	GATGGTTGC
CTCGATATC	GCGTCACCT	CGTTTACTA	GAGGAGCGT
CGAGACCGC	GGACACCTG	CGGGTCCTT	GACCGATGT
CGTTAGACA	GGTAACGGA	CGCTCACAC	GTACACCAA
CGGGGATCC	GGTTACCTT	CCATCCTGA	GTTCATACC
CGCGTTCTA	GGGGAGATC	CCTGATACT	GTGAATTTA
CCAGCTATT	GGCCAGAGG	CCGGAGTCA	GTGTGACAC
CCTGACAAA	GCAAGTCAC	CCCGATTGC	GTCGTGCTC
CCGCGATAG	GCATCTCGC	CAATACTAA	GTGGATAGG
AATTCAGGT	GGTGCGACC	TCGAAGGGA	AGCACCGTG
GCGCGCATA	GGTTGTTCT	TCGGCCACT	AGGTGGTAG
GCGGACTGC	GTAACAGTT	TCTGTACCG	AGTGTCACA
GCGGTTTAA	GTCCGTATT	TGCAGGTAC	ATAACTATG
GCTAGAGCT	GTCGTGAGT	TTCAAGCTC	ATCCTACCG
GCTGTCAGG	GTGGAACGA	AAACATACC	ACTACATCC
GCTTACAAG	GTTAACACG	AACGCAACC	TCCGACTAT
GCTTAGGAC	GTTTAGGTG	AAGCACTTG	GGCCATCAG
GGAATCGAG	TAACGCGCG	AATATATTA	GGTACAAGG
GGACGCCAC	TCACCTGAT	ACATACGGC	CTGTGGTAA
GGATAGTAA	TCCACCGCG	ACCCAGTTT	CGGACTCTC
GGCATTGTA	TCCACGTCA	ACGCATGGT	CCACGTGTC

Supplemental table 4: Sequencing primers used in this study for methylation analysis. Lower case letters denote the adaptor sequence to which the second round index primers bind.

Assay	Forward	Reverse	Size (bp)
FOXP3 TSDR	tgtaaaacgacggccagtTTGAATGGGGGATGTTTTTG	caggaaacagctatgaccAAAATATCTACCCTCTTCTCTTCCTC	209
CTLA4 exon 2	tgtaaaacgacggccagtGTGGTTTAGTTTGTTGTGGTATTG	caggaaacagctatgaccCTCCACCTTACAAATATAAAATCCC	315

Supplemental figure 1

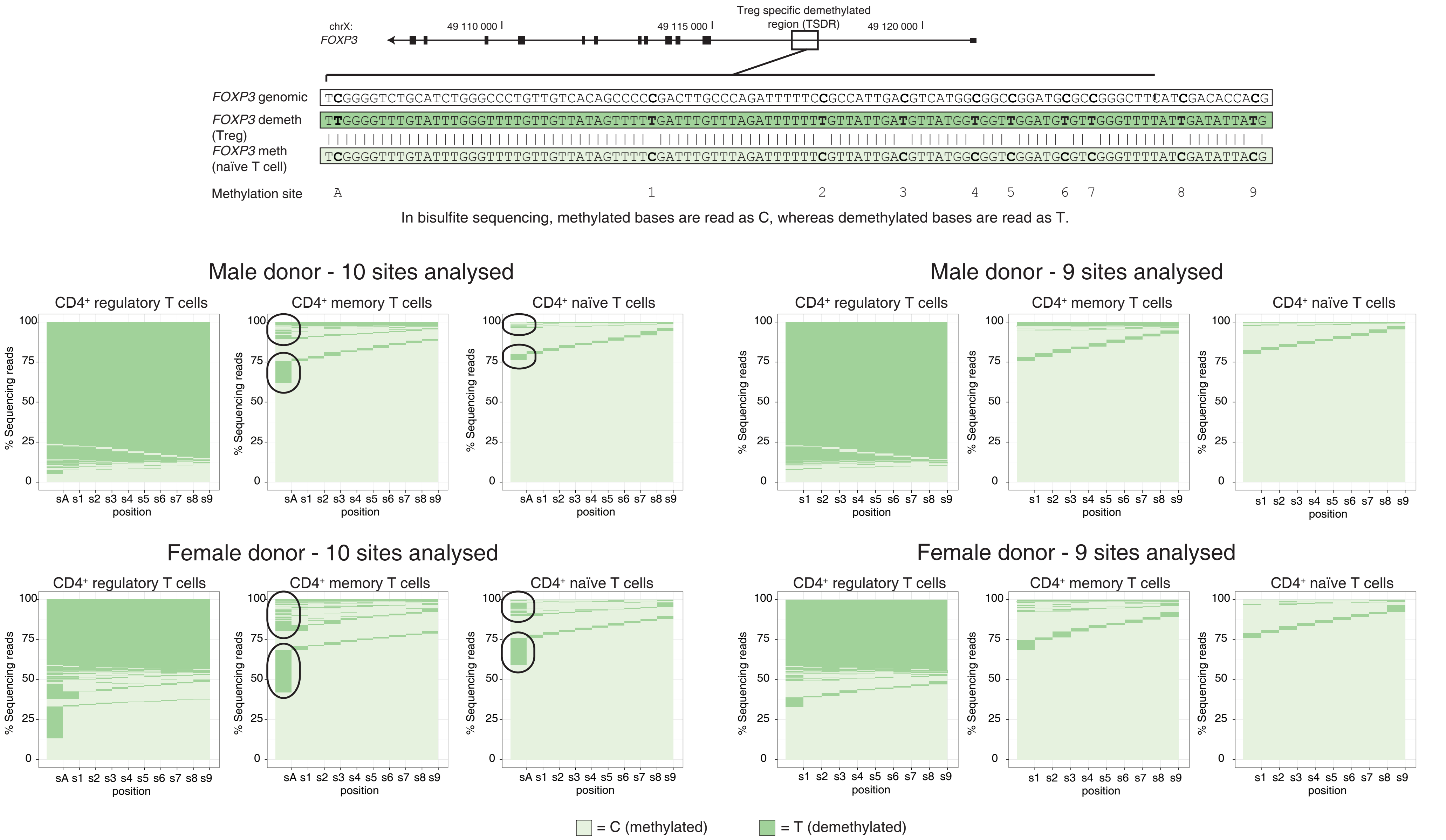

Supplemental figure 1: The *FOXP3* TSDR primers used target 10 CpG sites within the TSDR, nine of which have also been targeted by qPCR (sites numbered 1-9, Ref. 1). Site sA is not included as part of the analysis (black ovals, see Supplemental Methods section 1.14). Representative of two male and two females DNA samples.

1. Wieczorek, G. Cancer Res 2009. 69: 599-608.

Supplemental figure 2

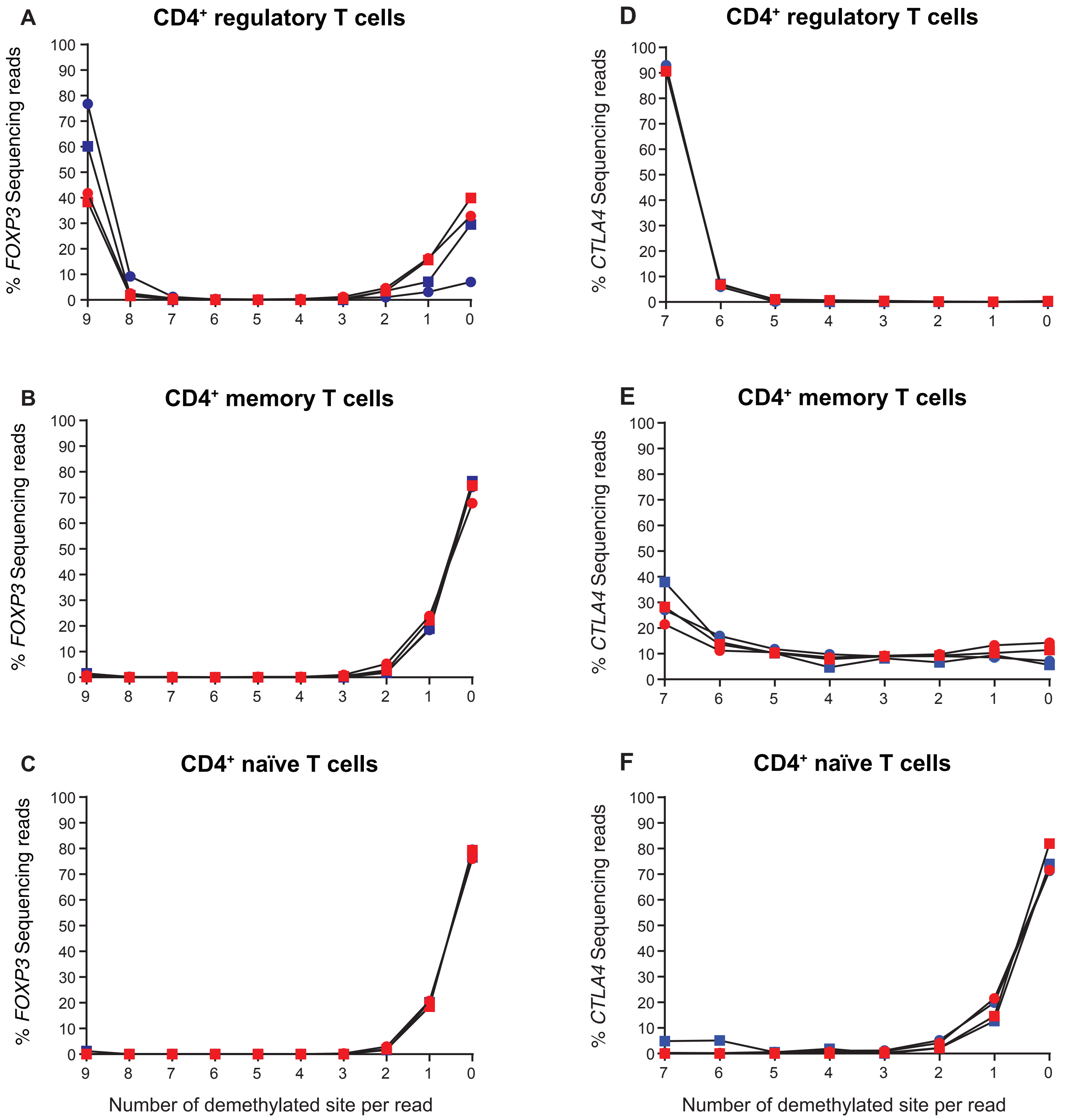

Supplemental figure 2: (A-C) The proportion of sequencing reads that contain the indicated number of demethylated (T) sites within each *FOXP3* read was measured from sorted Tregs and naïve and memory CD4⁺ T cells from two male (blue square and circle) and two female (red square and circle) donors (Supplemental Methods section 1.14). Supporting Information Table 1 shows percentages and read numbers. (D-F) The proportion of reads that contain the indicated number of demethylated (T) sites within each *CTLA4* read measured from sorted Tregs and naïve and memory CD4⁺ T cells from two male (blue square and circle) and two female (red square and circle) donors (Supplemental Methods section 1.14). Supporting Information Table 3 shows percentages and read numbers. The median of between 4 and 8 PCR replicates is displayed.

Supplemental Figure 3

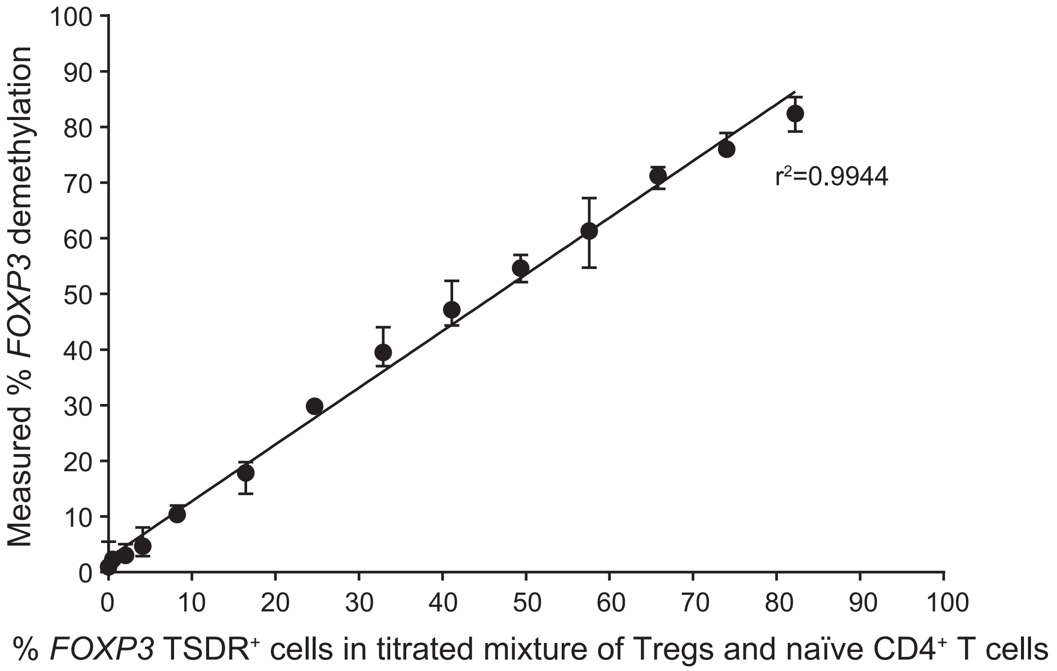

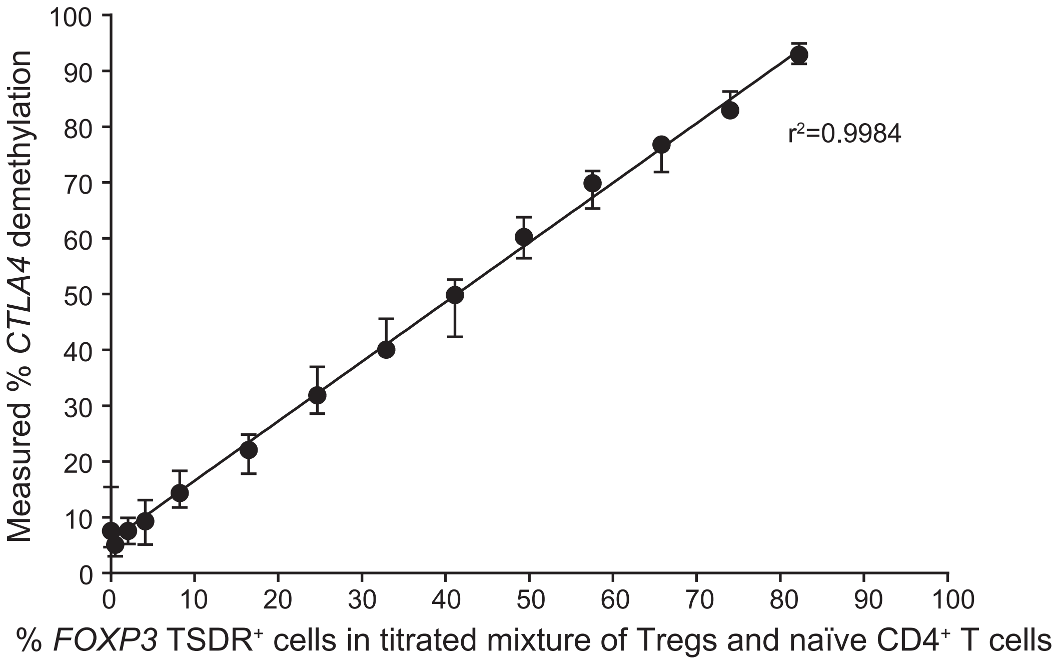

Supplemental figure 3: 20,000 cells were sorted from total CD4$^+$ T cells to generate differing ratios of Tregs and naïve CD4$^+$ T cells. Six PCR replicates were performed per sample (3300 cells per replicate) and the data shown are the median with range. The Treg sample was 82.3% demethylated at the *FOXP3* TSDR, therefore the highest point graphed on the X axis.

Supplemental figure 4

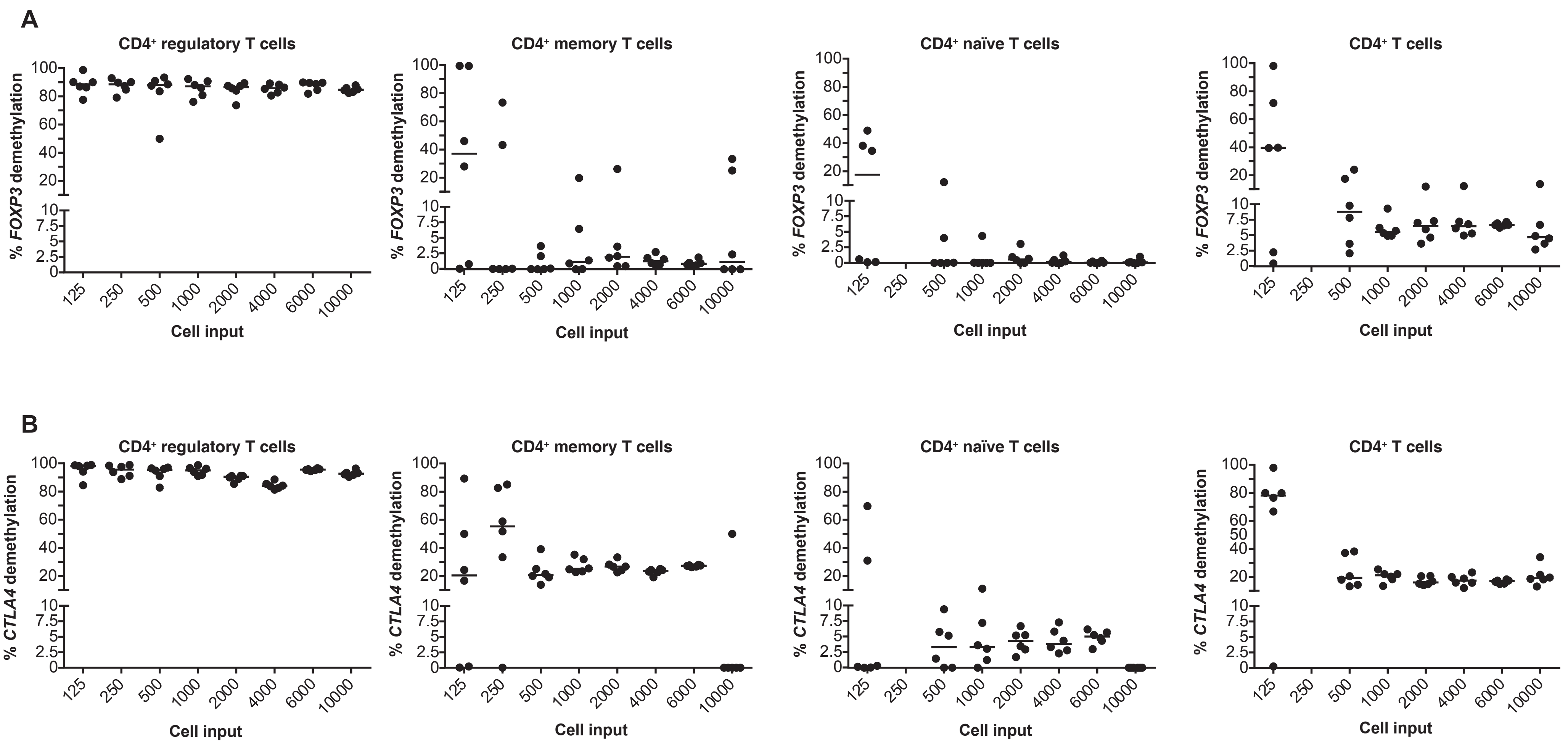

Supplemental figure 4. Tregs, memory, naïve and total CD4+ T cells were sorted and counted by the sorter and then titrated into the multiplexed bisulfite sequencing reaction. The x axis represents the number of cells used per PCR replicate. In each case six replicates were performed from each sorted cell population. Thus, the total number of cells collected for each input is six times the number shown on the x axis. Horizontal line represents the median. Cells were not collected for the naïve and CD4+ T cell 250 cell point.

Supplemental figure 5

A

Assay	Average conversion (%)	SD	Range (%)	Number of samples
FOXP3 TSDR	99.663	0.0827	99.2 - 100	1093
CTLA4 exon 2	99.713	0.0839	99.0 - 100	767

B

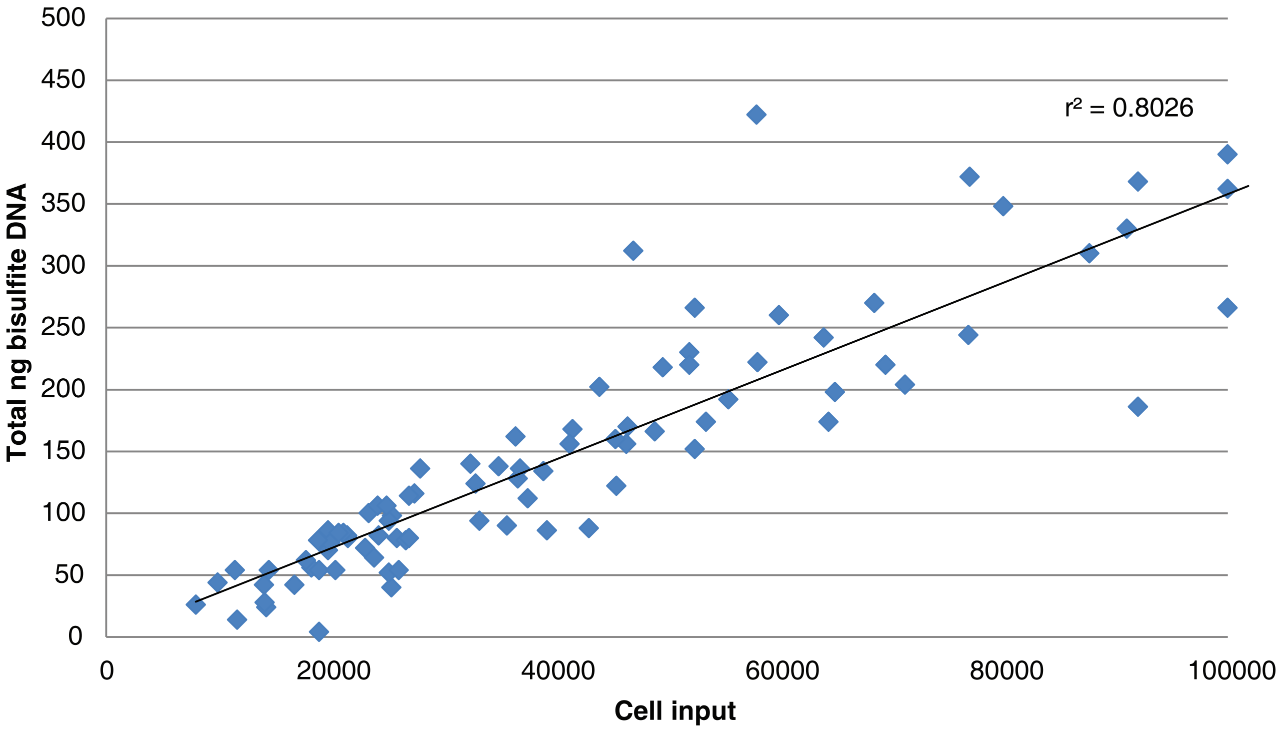

Supplemental figure 5: (A) Bisulfite conversion efficiencies for the *FOXP3* and *CTLA4* regions sequenced. (B) Relationship between cell input and quantity of bisulfite DNA extracted using the Qiagen Epitect Fast cell lysis kit (n=87).

Supplemental figure 6

```
human                1 TGGCCCAGCCTGCTGTGGTACTGGCCAGCAGCCGAGGCATCGCCAGCTTT      50
                       ||.||||.|||.|.|||||..||||.|||||||..||..|||||||||||
mouse                1 TGACCCAACCTTCAGTGGTGTTGGCTAGCAGCCATGGTGTCGCCAGCTTT      50

human               51 GTGTGTGAGTATGCATCTCCAGGCAAAGCCAC---TGAGGTCCGGGTGAC      97
                       ...|||||.|||.||.|..||..|||   |||   |||||||||||||||
mouse               51 CCATGTGAATATTCACCATCACACAA---CACTGATGAGGTCCGGGTGAC      97

human               98 AGTGCTTCGGCAGGC---TGACAGCCAGGTGACTGAAGTCTGTGCGGCAA     144
                       .|||||.||||||.|   |||   |||..|||||||.||||||||..|.|
mouse               98 TGTGCTGCGGCAGACAAATGA---CCAAATGACTGAGGTCTGTGCCACGA     144

human              145 CCT--ACATGATGGGGAATGAGT----TGACCTTCCTAGATGATTCCATC     188
                       |.|  |||     |.||| ||.|    ||..||||||||||.|..||.||
mouse              145 CATTCACA-----GAGAA-GAATACAGTGGGCTTCCTAGATTACCCCTTC     188

human              189 TGCACGGGCACCTCCAGTGGAAATCA-AGTGAACCTCACTATCCAAGGAC     237
                       ||||..||.||||..|.| ||||.|| ||||||||||||.||||||||||
mouse              189 TGCAGTGGTACCTTTAAT-GAAAGCAGAGTGAACCTCACCATCCAAGGAC     237

human              238 TGAGGGCCATGGACACGGGACTCTACATCTGCAAGGTGGA     277
                       ||||.||..|.|||||||||||.|||.|||||||||||||
mouse              238 TGAGAGCTGTTGACACGGGACTGTACCTCTGCAAGGTGGA     277
```

Supplemental figure 6: Homology between human and mouse *CTLA4* exon 2 sequences. The region identified as being differentially methylated between conventional T cells and regulatory T cells in mouse (1) was aligned to the human sequence. The CpG sites that are conserved between human and mouse are circled in red, and those that are unique to human are circled in blue. The pairwise sequence aligner, WATER (http://www.ebi.ac.uk), was used to perform the alignment.

1. Ohkura, N. Immunity 2012. 37: 785-799.

Supplemental figure 7

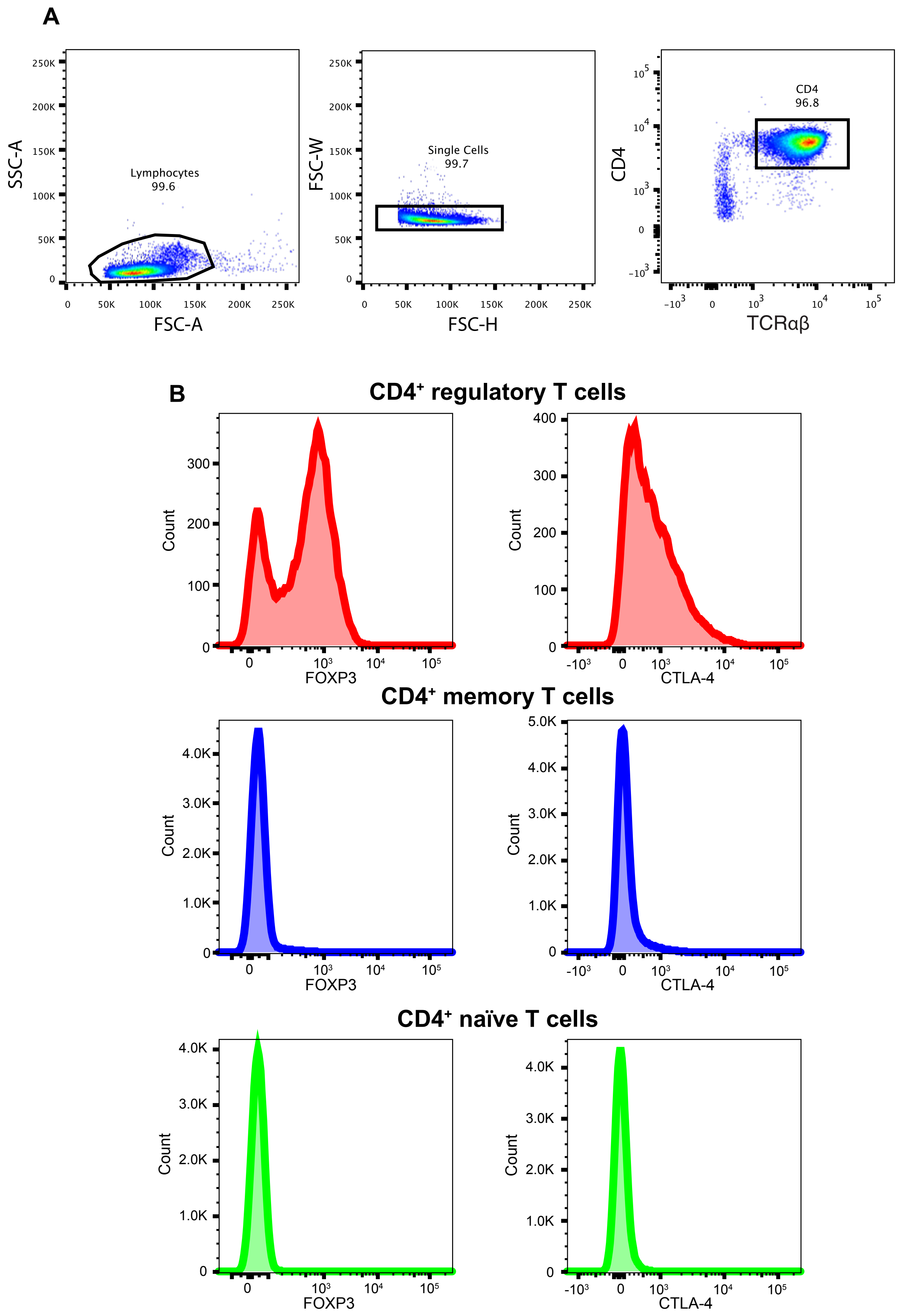

Supplemental figure 7. (A) The first steps in the gating strategy used to sort Tregs, memory and naïve CD4 T cells shown in Fig. 1. (B) Representative single parameter histograms showing the expression of FOXP3 and CTLA-4 in Tregs, memory and naïve CD4 T cells defined by the surface markers CD45RA, CD62L, CD127 and CD25 as shown in Fig. 1.

Supplemental figure 8: Workflow of the lab and bioinformatic protocol

LAB workflow

Sort cells

↓

One step cell lysis and bisulfite conversion

↓

1st PCR is gene specific, primers have adaptor sequence

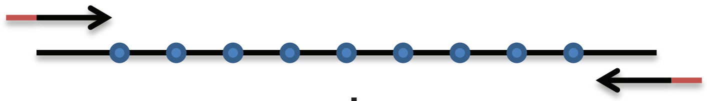

↓

2nd PCR adds index sequence that binds to adaptor sequence of 1st PCR primers

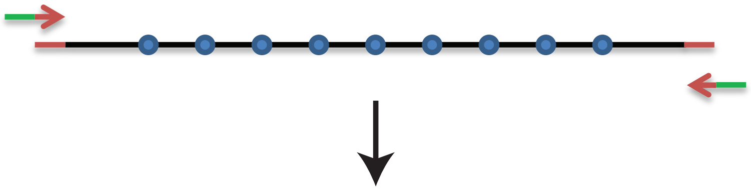

↓

Pool products at equimolar concentration for sequencing

↓

Sequence using Illumina MiSeq
300 bp paired end reads

Bioinformatic workflow

Read demultiplexing
(Novobarcode)

↓

Linker and poor quality sequence trimming
(Trimmomatic)

↓

Adaptor trimming
(Cutadapt)

↓

Stitching forward and reverse reads
(FLASH)

↓

Aligning to the reference sequence
(Bowtie 2)

↓

Indel adjusting and methylation site calling
(custom script)

↓

Data visualization
(R package: Methplot)
