# Supplementary material for: Epigenetic analysis of regulatory T cells using multiplex bisulfite sequencing
Source: Eur J Immunol. 2015 Oct 1;45(11):3200–3. doi: 10.1002/eji.201545646 (PMC4832369; doi:10.1002/eji.201545646)
Supplement: Supplementary file 1 — Epigenetic analysis of regulatory T cells using multiplex bisulfite sequencing [file EJI-45-3200-s001.pdf]

European Journal of Immunology

Supporting Information
for
DOI 10.1002/eji.201545646

Daniel B. Rainbow, Xin Yang, Oliver Burren, Marcin L. Pekalski, Deborah J. Smyth, Marcus D. R. Klarqvist, Christopher J. Penkett, Kim Brugger, Howard Martin, John A. Todd, Chris Wallace and Linda S. Wicker

Epigenetic analysis of regulatory T cells using multiplex bisulfite sequencing

© 2015 The Authors. *European Journal of Immunology* published by WILEY-VCH Verlag GmbH & Co. KGaA, Weinheim.

European Journal of Immunology

Supporting Information
for
DOI 10.1002/eji.201545646

Daniel B. Rainbow, Xin Yang, Oliver Burren, Marcin L. Pekalski, Deborah J. Smyth, Marcus D. R. Klarqvist, Christopher J. Penkett, Kim Brugger, Howard Martin, John A. Todd, Chris Wallace and Linda S. Wicker

Epigenetic analysis of regulatory T cells using multiplex bisulfite sequencing

© 2015 The Authors. *European Journal of Immunology* published by WILEY-VCH Verlag GmbH & Co. KGaA, Weinheim.

Epigenetic analysis of regulatory Tcells using multiplex bisulfite sequencing

Rainbow, Daniel; Yang, Xin; Burren, Oliver; Pekalski, Marcin; Smyth, Deborah; Klarqvist, Marcus; Penkett, Christopher; Martin, Howard; Todd, John; Wallace, Chris; Wicker, Linda

Correspondence: Prof. Wicker, Linda, Cambridge Institute for Medical Research, Cambridge, CB2 2XY, Tel:+44 (0) 1223 762816, Fax: +44 (0) 1223 762644

Review Timeline:	Submission date:	11-Mar-2015
	First Editorial decision:	17-Apr-2015
	Revision/s received:	07-Jul-2015
	Second Editorial decision:	11-Aug-2015
	Revision/s received:	14-Aug-2015
	Third Editorial decision:	17-Aug-2015
	Accepted:	01-Sep-2015

Handling Executive Committee member: Prof. Shimon Sakaguchi

Please note that the correspondence below does not include the standard editorial instructions regarding preparation and submission of revised manuscripts, only the scientific revisions requested and addressed.

First Editorial Decision – 17-Apr-2015

Dear Prof. Wicker,

Manuscript ID eji.201545646 entitled "Epigenetic analysis of regulatory Tcells using multiplex bisulfite sequencing" which you submitted to the European Journal of Immunology has been reviewed. The comments of the referees are included at the bottom of this letter.

A revised version of your manuscript that takes into account the comments of the referees will be reconsidered for publication. Should you disagree with any of the referees' concerns, you should address this in your point-by-point response and provide solid scientific reasons for why you will not make the requested changes.

You should also pay close attention to the editorial comments included below. **In particular, please

edit your figure legends to follow Journal standards as outlined in the editorial comments. Failure to do this will result in delays in the re-review process.**

Please note that submitting a revision of your manuscript does not guarantee eventual acceptance, and that your revision will be re-reviewed by the referees before a decision is rendered.

If the revision of the paper is expected to take more than three months, please inform the editorial office. Revisions taking longer than six months may be assessed by new referees to ensure the relevance and timeliness of the data.

Once again, thank you for submitting your manuscript to European Journal of Immunology and we look forward to receiving your revision.

Yours sincerely,
Katharina Schmidt

On behalf of
Prof. Shimon Sakaguchi

Dr. Katharina Schmidt
Editorial Office
European Journal of Immunology
e-mail: ejied@wiley.com

www.eji-journal.eu

Get the latest articles delivered directly to your desktop ! Register now for the free Wiley Alerting Service at
http://onlinelibrary.wiley.com/myprofile/alertManager

****

European Journal of Immunology

Reviewer: 1

Comments to the Author

The manuscript by Rainbow et al. developed a tool for assessing CpG methylation status of Foxp3 intron1 using NGS-based analysis. The method would be useful for detecting nTregs in humans and provide a new tool for assessing CpG methylation status of a large number of samples; however, there are some concerns regarding the tool established.

1. Accuracy would be the most important part for developing new tools. The authors should show the accuracy and reproducibility of the analysis. For example, analyzing the stepwise mixture of CpG methylated and demethylated DNAs (100:0, 90:10, 80:20, …etc) would be informative to show the accuracy. In addition, analyzing small number of cells might show high variability. It is also valuable to show the variability and reproducibility of samples obtained from a small number of cells.

2. In Fig. 1B, significant population of the completely CpG methylated reads are detected in regulatory T cell samples. It might be caused by a contamination of non-Treg cells within CD25+CD127low population. The purity of the fraction, such as Foxp3high population, would be shown.

3. In addition to FOXP3 intron1 and CTLA4 exon2, the Treg-specific demethylated regions are also detected within Eos and Helios genes. If FOXP3 and CTLA4 loci were not suitable for the evaluation, the authors would propose the most suitable locus for detecting nTregs.

Reviewer: 2

Comments to the Author

In their technical comment, Rainbow et al. describe a NGS-based method to assess the methylation status of FOXP3 TSDR and CTLA4 (region in exon 2). Using this method, the authors have analyzed the FOXP3 TSDR and CTLA4 methylation status in three CD4+ T cell subsets (naive, effector/memory and regulatory) isolated from healthy donors. They could confirm published results for FOXP3 TSDR, while they observed a heterogeneous demethylation pattern for CTLA4 in effector/memory cells, in contrast to what has been previously published for the murine system. Although the overall topic is of interest, the

manuscript in its present form provides only little increment over already published data.

Specific points:

1) In contrast to what has been stated in the manuscript, the methylation status of the TSDR does not allow to discriminate between thymus-derived and peripherally induced Tregs. Instead, a demethylated TSDR identifies Tregs with stable FOXP3 expression.
2) Previously published data have already analyzed the methylation status at each CpG site on a single piece of DNA (e.g. Kim et al., J Exp Med 204:1543 or Lal et al., J Immunol 182:259). These papers were not cited by Rainbow et al.
3) The authors did neither report the sort purity nor checked FOXP3 and CTLA-4 expression in sorted CD4+ T cell subsets.
4) It is neither obvious from the methods section (1.4 and 1.6) nor from the data depicted in Suppl. Figure 5 whether the whole experimental procedure can be performed with as low as 2000 cells as starting material.

Reviewer: 3

Comments to the Author
The data presented in this technical report is solid. Although methylation analysis of FOXP3 and CTLA4 enhancers have been repeatedly reported, the features of using low cell number and multiplexing is of value. Some improvement for clarity is needed.

1. the methplots used throughout the manuscript is confusing. What exactly does Y axis represent? If the numbers represent proportion (%?) of reads, what does it mean that all the lines between 100 and 25 have identical pattern of solid dark green in Figure 1B left panel? Please provide better explanation for the Y axis.

2. what is the lowest number of cells that can be used in this assay to have reliable results?

3. The finding that memory T cells have demethylated CTLA4 exon 2 is interesting. The authors stated that this finding is in contrast to the data using mouse cells in a previous publication ref 11 (last line,

page 4). This is not entirely correct. The previous publication showed that T cells activated short-term in vitro have methylated pattern at CTLA4 exon 2, but demthylated with chronic activation. the authors should correct their statement to say that human memory T cells resemble chronically activated mouse T cells.

First Revision – authors' response – 07-Jul-2015

Reviewer: 1

>

> Comments to the Author

> The manuscript by Rainbow et al. developed a tool for assessing CpG methylation status of Foxp3 intron1 using NGS-based analysis. The method would be useful for detecting nTregs in humans and provide a new tool for assessing CpG methylation status of a large number of samples; however, there are some concerns regarding the tool established.

>

> 1. Accuracy would be the most important part for developing new tools. The authors should show the accuracy and reproducibility of the analysis. For example, analyzing the stepwise mixture of CpG methylated and demethylated DNAs (100:0, 90:10, 80:20, …etc) would be informative to show the accuracy. In addition, analyzing small number of cells might show high variability. It is also valuable to show the variability and reproducibility of samples obtained from a small number of cells.

We welcome the opportunity to add data addressing these questions. The accuracy of the FOXP3 TSDR and the CTLA4 exon 2 assays (r2 of 0.9944 and 0.9984, respectively) is shown in Supporting Information Fig. 3 where mixtures of Tregs and naïve CD4+ T cells were prepared using the cell sorter. This experiment also demonstrates the high fidelity of our cell sorting. We have added Supporting Information Fig. 4, which shows a cell input titration of four different cell populations for both the FOXP3 TSDR and the CTLA4 exon 2 region. Scatter plots showing each replicate for each cell input are graphed. Although we conservatively recommend 2000 cells (5 ng DNA) per replicate and six replicates, these supplemental data show that even lower cell numbers may provide valid data if replicates are consistent. We also show multiplex data with the FOXP3 and CTLA4 gene regions but state on page 4 that we have successfully multiplexed six regions with 2000-4000 cells per replicate.

> 2. In Fig. 1B, significant population of the completely CpG methylated reads are detected in regulatory T cell samples. It might be caused by a contamination of non-Treg cells within CD25+CD127low population. The purity of the fraction, such as Foxp3high population, would be shown.

Since this was a technical communication focused on the development of the NGS-based assay, we opted for sorting Tregs using cell surface markers rather than FOXP3 primarily due to the reduction in the yields of Tregs caused by the additional washes required for the intracellular staining protocol. The fact that CD4+ CD25+ CD127−/low cells are highly enriched for FOXP3+ cells (86.6%, range 67.4-93.6 for 10 donors) that mediate suppression was first defined by Bluestone and colleagues (JEM 2006 - now added as a reference in the paper) and we and other investigators have confirmed their observation. We have added this clarification to Supporting Information section 1.2.

We are currently utilising the NGS-based assay described in our manuscript to analyse DNA from cells sorted based on FOXP3 and HELIOS intracellular staining in addition to surface markers and we are observing the expected increases in the percentage of TSDR reads that are demethylated as compared to CD4+ CD25+ CD127−/low cells sorted based only on cell surface markers. We believe these results are outside the scope of the current technical comment and have not added them to the manuscript.

>

> 3. In addition to FOXP3 intron1 and CTLA4 exon2, the Treg-specific demethylated regions are also detected within Eos and Helios genes. If FOXP3 and CTLA4 loci were not suitable for the evaluation, the authors would propose the most suitable locus for detecting nTregs.

>

We have looked for orthologous regions in human to the Treg-specific demethylated regions identified in mouse by Ohurka et al., but it was only the CTLA4 exon 2 region that showed good conservation between human and mouse thereby justifying the investigation of this particular region. We have now modified the text to indicate our rationale for highlighting the CTLA4 analysis (Page 5). Our primary goal in showing the data from the CTLA4 gene was to demonstrate an example of multiplexing using the NGS-based assay. We will report on other differentially demethylated regions in future publications, but to date, we have not found any region with Treg specificity equal to that of the FOXP3 TSDR for human CD4+ T cells.

>

> Reviewer: 2

>

> Comments to the Author

> In their technical comment, Rainbow et al. describe a NGS-based method to assess the methylation status of FOXP3 TSDR and CTLA4 (region in exon 2). Using this method, the authors have analyzed the FOXP3 TSDR and CTLA4 methylation status in three CD4+ T cell subsets (naive, effector/memory and regulatory) isolated from healthy donors. They could confirm published results for FOXP3 TSDR, while they observed a heterogeneous demethylation pattern for CTLA4 in effector/memory cells, in contrast to what has been previously published for the murine system. Although the overall topic is of interest, the manuscript in its present form provides only little increment over already published data.

We agree there are methods available to assess the methylation status of the FOXP3 TSDR, however they all have significant limitations. Methods measuring the difference in peak height of Sanger sequence traces produce an average methylation per site and qPCR based assays produce an average methylation value across several CpG sites, whereas our method provides information about each CpG site on individual alleles of DNA. PCR cloning and sequencing is low throughput with less than 50 copies of DNA usually assessed. Additionally, our method is applicable to any region of interest (without the need to find methylation specific primers) as shown with the example of the CTLA4 exon 2 region, which was performed in a multiplex assay. Current methods to validate whole genome NGS-based bisulfite sequencing still routinely use PCR cloning and Sanger sequencing (for example Okae H, Plos Genetics 2014; Stevens M, Genome Research 2013) to confirm results. Therefore, we believe that a detailed, transparent publication which highlights the advantages of using next-generation sequencing for targeted analysis of methylation would be of general interest.

>

> Specific points:

>

> 1) In contrast to what has been stated in the manuscript, the methylation status of the TSDR does not allow to discriminate between thymus-derived and peripherally induced Tregs. Instead, a demethylated TSDR identifies Tregs with stable FOXP3 expression.

We agree with the reviewer that the frequently heard and read statement indicating that cells demethylated at the TSDR are derived from the thymus is an overstatement and we have removed this concept from the manuscript. We have also removed the term “natural Treg” from the manuscript since

it implies that the Tregs are derived from the thymus. As suggested by the reviewer we now describe Tregs as follows: “A subset of regulatory CD4+ T cells (Tregs) is characterised by the stable constitutive expression of the transcription factor FOXP3.”

> 2) Previously published data have already analyzed the methylation status at each CpG site on a single piece of DNA (e.g. Kim et al., J Exp Med 204:1543 or Lal et al., J Immunol 182:259). These papers were not cited by Rainbow et al.

We have added the following text to page 2 of the manuscript and referenced three papers which use the PCR cloning and sequencing method. We have included the Kim et al. reference suggested by the reviewer, but not the Lal et al. reference since this study analyses the FOXP3 upstream enhancer region rather than the TSDR.

“Although several studies have used a PCR cloning and sequencing method to assess the methylation of each CpG site within the TSDR, this is a laborious method and normally less than 50 clones per sample are analysed [3, 9, 10].”

> 3) The authors did neither report the sort purity nor checked FOXP3 and CTLA-4 expression in sorted CD4+ T cell subsets.

We now state in the Supporting Information that our level of purity from sorting on the FACSAria III ranges from 98.0% to 99.9%.

In regard to FOXP3 and CTLA-4 protein expression in sorted CD4+ T cell subsets, since this was a technical comment focused on the development of the NGS-based assay, we opted for sorting Tregs using cell surface markers rather than FOXP3 or CTLA-4 primarily due to the reduction in the yields of Tregs caused by the additional washes required for the intracellular staining protocol. The fact that CD4+ CD25+ CD127−/low cells are highly enriched for FOXP3+ cells (86.6%, range 67.4-93.6 for 10 donors) that mediate suppression was first defined by Bluestone and colleagues (JEM 2006—now added as a reference in the paper) and we and other investigators have confirmed their observation. We have added this clarification to Supporting Information section 1.2. Similarly, a strong correlation between the levels of FOXP3 and CTLA-4 in Tregs has been demonstrated by Miyara et al. (Immunity 2009. 30:

European Journal of Immunology

899-911) and we have confirmed these findings but believe that showing these data is outside the scope of the current technical comment and have not included them. Since demethylation of exon 2 in human CTLA4 is not restricted to Tregs, we are characterising the demethylation patterns in memory and naïve effector CD4+ T cell subsets for a future publication. Interestingly, Miyara et al. showed that there is a population of CD4+ T cells that are FOXP3−, but express CTLA-4 and are CD45RA−, which may represent the portion of the memory population that we have observed is fully demethylated at CTLA4.

> 4) It is neither obvious from the methods section (1.4 and 1.6) nor from the data depicted in Suppl. Figure 5 whether the whole experimental procedure can be performed with as low as 2000 cells as starting material.

>

As detailed in our reply to Reviewer 1's first question, we have added Supporting Information Fig. 4 which shows a cell input titration of four different cell populations for both the FOXP3 TSDR and the CTLA4 exon 2 region. Scatter plots showing each replicate for each cell input are graphed. Although we conservatively recommend 2000 cells (5 ng DNA) per replicate and six replicates, these supplemental data show that even lower cell numbers may provide valid data if replicates are consistent. We also show multiplex data with the FOXP3 and CTLA4 gene regions but state on page 4 that we have successfully multiplexed six regions with 2000-4000 cells per replicate.

> Reviewer: 3

>

> Comments to the Author

> The data presented in this technical report is solid. Although methylation analysis of FOXP3 and CTLA4 enhancers have been repeatedly reported, the features of using low cell number and multiplexing is of value. Some improvement for clarity is needed.

>

> 1. the methplots used throughout the manuscript is confusing. What exactly does Y axis represent? If the numbers represent proportion (%?) of reads, what does it mean that all the lines between 100 and 25 have identical pattern of solid dark green in Figure 1B left panel? Please provide better explanation for the Y axis.

>

We have revised the legend to Figure 1 and the title of y axis on the methplots to reduce confusion. The

Figure legend in part now reads, “The x axis shows the nine methylation sites analysed and each row indicates the methylation status of one copy of the sequenced TSDR. Light green represents a C (methylated) and dark green represents a T (demethylated). The y axis is the percentage of sequencing reads for each methylation/demethylation pattern.” For example, in the left hand plot in figure 1B a Treg sample has been analysed and just over 75% of the reads are fully demethylated (dark green across each row from ~25% to 100%), we then see approximately 10% of reads which have a mosaic methylation pattern with 1 or more CpG sites methylated and the remainder demethylated. The final 15% of reads are light green across the row, which means they are fully methylated, or have only one or two sites demethylated. To make the plots less “cluttered” the total number of reads for each analysis is now detailed in Supporting Information Table 1 and Table 3.

> 2. what is the lowest number of cells that can be used in this assay to have reliable results?

As detailed in our reply to Reviewer 1’s first question, we have added Supporting Information Fig. 4 which shows a cell input titration of four different cell populations for both the FOXP3 TSDR and the CTLA4 exon 2 region. Scatter plots showing each replicate for each cell input are graphed. Although we conservatively recommend 2000 cells (5 ng DNA) per replicate and six replicates, these supplemental data show that even lower cell numbers may provide valid data if replicates are consistent. We also show multiplex data with the FOXP3 and CTLA4 gene regions but state on page 4 that we have successfully multiplexed six regions with 2000-4000 cells per replicate.

> 3. The finding that memory T cells have demethylated CTLA4 exon 2 is interesting. The authors stated that this finding is in contrast to the data using mouse cells in a previous publication ref 11 (last line, page 4). This is not entirely correct. The previous publication showed that T cells activated short-term in vitro have methylated pattern at CTLA4 exon 2, but demthylated with chronic activation. the authors should correct their statement to say that human memory T cells resemble chronically activated mouse T cells.
>

Owing to the word limits of a Technical Comment, a proper discussion of the methylation status of CTLA4 exon 2 in human versus mouse T cells is not possible; therefore, we have eliminated the phrase, “In contrast to the results from mouse Ctla4” from the manuscript and focus on our results from human

cells. Since we have found that demethylation of exon 2 in human CTLA4 is not restricted to Tregs, we are characterising the demethylation patterns in memory and naïve effector CD4+ T cell subsets for a future publication. Interestingly, Miyara et al. (Immunity 2009. 30: 899-911) showed that there is a population of human CD4+ T cells that are FOXP3−, but express CTLA-4 and are CD45RA−, which may represent the portion of the memory population that we have observed is fully demethylated at CTLA4.

Second Editorial Decision - 11-Aug-2015

Dear Prof. Wicker,

Thank you for submitting your revised manuscript ID eji.201545646.R1 entitled "Epigenetic analysis of regulatory Tcells using multiplex bisulfite sequencing" to the European Journal of Immunology. Your manuscript has been re-reviewed and the comments of the referees are included at the bottom of this letter.

Although the referees have recommended publication, some minor revisions to your manuscript have been requested. Therefore, I invite you to respond to the comments of the referees and revise your manuscript accordingly.

You should also pay close attention to the editorial comments included below. **In particular, please edit your figure legends to follow Journal standards as outlined in the editorial comments. Failure to do this will result in delays in the re-review process.**

If the revision of the paper is expected to take more than three months, please inform the editorial office. Revisions taking longer than six months may be assessed by new referees) to ensure the relevance and timeliness of the data.

Once again, thank you for submitting your manuscript to European Journal of Immunology. We look forward to receiving your revision.

Yours sincerely,

Katharina Schmidt

on behalf of
Prof. Shimon Sakaguchi

Dr. Katharina Schmidt
Editorial Office
European Journal of Immunology
e-mail: ejied@wiley.com

www.eji-journal.eu

Get the latest articles delivered directly to your desktop ! Register now for the free Wiley Alerting Service at
http://onlinelibrary.wiley.com/myprofile/alertManager

****

Reviewer: 1

Comments to the Author
The revision is satisfactory for this reviewer.

Reviewer: 3

Comments to the Author
The authors have adequately addressed my comments. There is a large body of supplemental data scattered in many files, which are hard to track. It would be better to have some of them consolidated into one. Data Table 4 is not needed.

Reviewer: 2

Comments to the Author

In the revised version of their technical comment, Rainbow et al. have addressed most of my specific concerns and have better explained the novelty of their data. Still, some minor points need to be addressed before allowing publication of this manuscript:

1) The authors should depict the existing data on FOXP3 and CTLA-4 expression in sorted CD4+ T cell subsets.
2) Figure 1, 2 and S1: The number of reads depicted in the respective Figures needs to be mentioned.
3) Figure 1D is redundant to part of Figure S1 and can be deleted.
4) Figure S3: The description of the x-axis needs to be modified to: % of Tregs (not FOXP3 TSDR+ cells) in titrated mixtures of Tregs and naive CD4+ T cells.
5) Page 3, line 25: delete „effector“.

Third Editorial Decision – 17-Aug-2015

Dear Prof. Wicker,

It is a pleasure to provisionally accept your manuscript entitled "Epigenetic analysis of regulatory Tcells using multiplex bisulfite sequencing" for publication in the European Journal of Immunology. For final acceptance, please follow the instructions below and return the requested items as soon as possible as we cannot process your manuscript further until all items listed below are dealt with.

Please note that EJI articles are now published online a few days after final acceptance (see Accepted Articles: http://onlinelibrary.wiley.com/journal/10.1002/(ISSN)1521-4141/accepted). The files used for the Accepted Articles are the final files and information supplied by you in Manuscript Central. You should therefore check that all the information (including author names) is correct as changes will NOT be permitted until the proofs stage.

We look forward to hearing from you and thank you for submitting your manuscript to the European Journal of Immunology.

Yours sincerely,
Katharina Schmidt

European Journal of Immunology

on behalf of

Prof. Shimon Sakaguchi

Dr. Katharina Schmidt

Editorial Office

European Journal of Immunology

e-mail: ejied@wiley.com

www.eji-journal.eu
